# Discovery and Characterization of the *ddx41* Gene in Atlantic Salmon: Evolutionary Implications, Structural Functions, and Innate Immune Responses to *Piscirickettsia salmonis* and *Renibacterium salmoninarum* Infections

**DOI:** 10.3390/ijms25126346

**Published:** 2024-06-08

**Authors:** Alejandro J. Yañez, Claudia A. Barrientos, Adolfo Isla, Marcelo Aguilar, Sandra N. Flores-Martin, Yassef Yuivar, Adriana Ojeda, Pablo Ibieta, Mauricio Hernández, Jaime Figueroa, Rubén Avendaño-Herrera, Marcos Mancilla

**Affiliations:** 1 Laboratorio de Diagnóstico y Terapia, Facultad de Ciencias, Universidad Austral de Chile, Valdivia 5090000, Chile; claud.barrientosman@gmail.com (C.A.B.); adolfoisla@gmail.com (A.I.); marcelo.aguilarcart@gmail.com (M.A.); sandra_uach_2008@hotmail.com (S.N.F.-M.); 2 Interdisciplinary Center for Aquaculture Research (INCAR), Concepción 4030000, Chile; jefigueroa@uach.cl (J.F.); reavendano@yahoo.com (R.A.-H.); 3 Departamento de Ciencias Básicas, Facultad de Ciencias, Universidad Santo Tomas, Valdivia 5090000, Chile; 4 ADL Diagnostic Chile, Sector la Vara, Puerto Montt 5480000, Chile; yyuivar@adldiagnostic.cl (Y.Y.); aojeda@adldiagnostic.cl (A.O.); 5 TEKBios Ltda, Camino Pargua Km 8, Maullín 5580000, Chile; pablo.ibieta@tekbios.cl; 6 Division of Biotechnology, MELISA Institute, San Pedro de la Paz 4133515, Chile; mhernandez@melisainstitute.org; 7 Laboratorio de Biología Molecular de Peces, Instituto de Bioquímica y Microbiología, Facultad de Ciencias, Universidad Austral de Chile, Valdivia 5090000, Chile; 8 Laboratorio de Patología de Organismos Acuáticos y Biotecnología Acuícola, Facultad de Ciencias de la Vida, Universidad Andrés Bello, Viña del Mar 2520000, Chile

**Keywords:** *ddx41* gene, functional gene expression, structural–functional analysis, innate immune response, Atlantic salmon–pathogen communication, bacterial infection

## Abstract

The innate immune response in *Salmo salar*, mediated by pattern recognition receptors (PRRs), is crucial for defending against pathogens. This study examined DDX41 protein functions as a cytosolic/nuclear sensor for cyclic dinucleotides, RNA, and DNA from invasive intracellular bacteria. The investigation determined the existence, conservation, and functional expression of the *ddx41* gene in *S. salar*. In silico predictions and experimental validations identified a single *ddx41* gene on chromosome 5 in *S. salar*, showing 83.92% homology with its human counterpart. Transcriptomic analysis in salmon head kidney confirmed gene transcriptional integrity. Proteomic identification through mass spectrometry characterized three unique peptides with 99.99% statistical confidence. Phylogenetic analysis demonstrated significant evolutionary conservation across species. Functional gene expression analysis in SHK-1 cells infected by *Piscirickettsia salmonis* and *Renibacterium salmoninarum* indicated significant upregulation of DDX41, correlated with increased proinflammatory cytokine levels and activation of *irf3* and interferon signaling pathways. In vivo studies corroborated DDX41 activation in immune responses, particularly when *S. salar* was challenged with *P. salmonis*, underscoring its potential in enhancing disease resistance. This is the first study to identify the DDX41 pathway as a key component in *S. salar* innate immune response to invading pathogens, establishing a basis for future research in salmonid disease resistance.

## 1. Introduction

In the intricate interaction between a host and a pathogen, numerous mechanisms are involved, with the innate immune response being the primary line of defense in fishes against invading pathogens [1,2,3]. The mechanisms driving this defense system have attracted considerable attention, especially with the discovery of pattern recognition receptors (PRRs). These receptors play a crucial role in recognizing pathogen-associated molecular patterns (PAMPs) and initiating the production of proinflammatory cytokines or interferons [4]. An array of PRRs reside within host cells, strategically positioned in subcellular compartments, effectively serving as detectors that alert the host to potential threats [5]. The activation of these host defense mechanisms triggers complex immune signaling pathways, establishing lasting immunity against pathogens [4,6]. Extensive studies have analyzed the functional properties and specific signaling pathways of gene expression in mammals and their potential in biotherapeutic applications [7,8]. However, the search for homologous proteins in salmonids, particularly *Salmo salar*, underscores the imperative for a more comprehensive exploration of innate immune system components in this species.

The interaction between pathogens and hosts reveals an wide array of strategies employed by microorganisms to manipulate host cell functions [9]. One such strategy involves the utilization of secondary messengers like cyclic-di-GMP (c-di-GMP) or cyclic-di-AMP (c-di-AMP) by diverse microorganisms [10,11]. These secondary messengers modulate essential host cell functions, prompting eukaryotic cells to develop mechanisms to sense intracellular bacteria through specialized cytosolic sensor proteins, specifically designed for bacterial DNA, RNA, and cyclic metabolites [12,13,14]. Among these cytosolic sensors, DEAD-box polypeptide 41 (DDX41) has emerged as a key player with diverse roles in innate immunity and diseases [15,16], functioning as a direct PRR for cyclic dinucleotides in both mouse and human cells [11,17]. This protein is conserved from bacteria to mammals and is characterized by a specific amino acid motif with Asp-Glu-Ala-Asp or the DEAD motif [18]. The recognition of cyclic dinucleotides, particularly c-di-AMP secreted by pathogens like *Listeria monocytogenes*, activates the cytosolic surveillance pathway, detecting a spectrum of pathogens including bacteria, viruses, and protozoa [10].

In this sense, DDX41 plays a crucial role in the IRF3 signaling pathway and displays a special capacity to recognize various ligands, encompassing double-stranded RNA, DNA, and cyclic dinucleotides through its DEAD domain [19].

The significance of DDX41 has surged in recent years, especially its role in diverse innate immune responses against pathogens. Functionally, DDX41 operates via the IRF3 pathway, directly binding to DNA and STING through its DEAD domain [20]. Upon infection, the release of dsDNA or c-di-GMP by viruses or bacteria acts as a PAMP to stimulate signaling pathways that sequentially activate other molecules, such as interleukin-1β (IL-1β), tumor necrosis factor-α (TNFα) and interferon-γ (IFNγ), enhancing the host’s innate response [20,21,22]. In various fish species such as *Paralichthys olivaceus* [23], *Danio rerio* [21], *Epinephelus coioides* [24], *Oreochromis niloticus* [25] and *Siniperca chuatsi* [26], DDX41 has been identified, demonstrating its evolutionary conservation and significance in fish immunity. In *P. olivaceus*, the induction of *ddx41* gene expression was observed following DNA virus infection [23]. Similarly, in *D. rerio*, the role of the *ddx41* gene in antibacterial innate immunity against infections with *Aeromonas hydrophila* and *Edwardsiella tarda* was identified [21]. Studies conducted on these species have shown their involvement in triggering interferon-mediated antiviral and inflammatory responses upon viral or bacterial infections. The exploration of DDX41 protein homologues in salmonids, with a particular focus on *S. salar*, is notably absent and lacks detailed characterization. This deficiency underscores the urgent necessity for a thorough investigation into the innate immune pathways mediated by the existence of the *ddx41* gene within *S. salar*, to elucidate their structural and functional roles and evolutionary significance.

*S. salar* is a crucially important salmonid in the world aquaculture industry [27], but faces significant threats from bacterial diseases, primarily caused by *Piscirickettsia salmonis* and *Renibacterium salmoninarum* [28]. These Gram-negative and Gram-positive intracellular bacteria pose substantial challenges, causing septicemic diseases with high mortalities in salmonid populations [28,29]. The ability of these pathogens to invade, survive, and multiply inside the host’s cell or tissues is considered a key aspect of their virulence [27]. The significance of DDX41 as an intracellular PRR, and its involvement in eliciting immune responses against bacterial infections in *S. salar*, is the primary focus and motivation of this study [10,21]. Understanding the mechanisms underlying the functional responses of immune gene expression to infections in *S. salar* against these pathogens significantly contributes to preserving salmon health [27].

Hence, this study aims to elucidate the existence, structure, and evolutionary significance of the *ddx41* gene orthologs in the *S. salar* immune system. It explores the gene’s phylogenetic relationships and examines the conservation of structural and functional domains throughout evolution. Additionally, this study assesses *ddx41* gene expression in response to infections by *P. salmonis* and *R. salmoninarum* in Salmon Head Kidney-1 (SHK-1) cell lines and its in vivo response in *S. salar* following challenges with *P. salmonis*. This research underscores the importance of characterizing key innate immune genes that function as cytosolic sensors, crucial for modulating immune pathways during host–pathogen interactions. By advancing our understanding of these genes, we aim to identify novel targets for enhancing disease control in aquaculture.

## 2. Results

### 2.1. In Silico and Experimental Characterization of ddx41 Gene and Protein of S. salar

**Figure 1:**
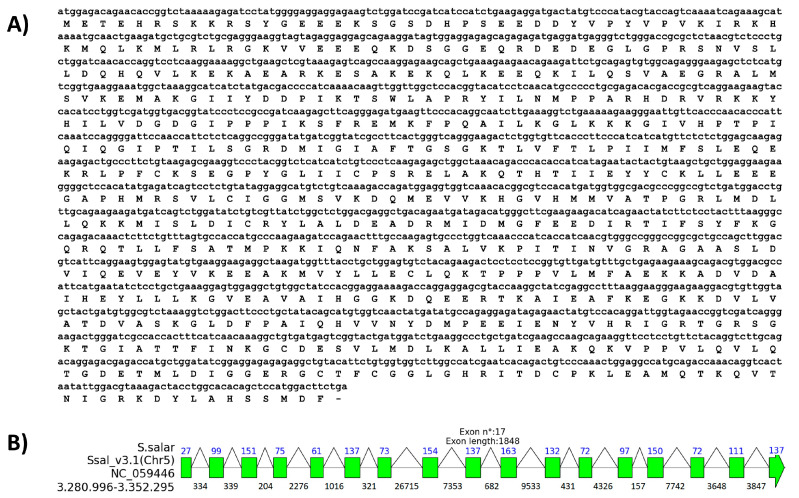
Description of *ddx41* sequence of *S. salar*. (**A**) Nucleotide and amino acid sequence; (**B**) chromosome localization indicates intron and exons.

**Prediction of the existence of an orthologous gene.** Initially, we retrieved the predicted coding sequence by conducting a BLAST search with the human *ddx41* gene against salmonid taxa in the NCBI database. The results revealed only one sequence aligning within the *S. salar* genome, indicating the existence of a single homolog to the human gene (Table 1). The sequence of the DEAD box helicase 41 (*ddx41*) gene in the *S. salar* genome can be obtained through the GenBank database (Accession number NM001140327.1) (Figure 1A). The genomic analysis clarified that the described gene architecture is situated on chromosome 5, condensed within a 71.3 megabase (Mb) region. This configuration consists of 17 exons combined by 16 introns, with a coding sequence extending over 1848 base pairs (bp), ending in the translation of a protein composed of 615 amino acids (Figure 1A,B). The gene organization visualization of the *S. salar ddx41* gene highlights three introns over 5000 nucleotides. In *S. salar*, *ddx41* is between 3.28 and 3.352 Mbps (Figure 1B), with respect to the reference genome collected in Ssal_v3.1 (NCBI accession number GCF_905237065.1), as the most recent version. Comparison of sequence using BLAST, revealed that *R. norvegicus* and *G. gallus* share higher identity percentages with *S. salar ddx41* at both nucleotide and protein sequences. Moreover, the results showed that the nucleotide and amino acid sequence are closely related to *Homo sapiens ddx41*, with a 78.61% identity and a 91% query coverage in nucleotide analysis (Table 1). Protein BLAST results further supported this closeness, demonstrating an 83.92% identity with 100% query coverage. This similarity is more pronounced at the protein level, with only minor variations in identity percentages from nucleotide to protein analyses. The comparative analysis underscores a closer evolutionary relationship between *S. salar* DDX41 and *H. sapiens* DDX41, highlighting the conservation of *ddx41* function across diverse species, from fish to humans (Table 1). Furthermore, the deduced amino acid sequence of DDX41 from *S. salar* was aligned with the *ddx41* sequences from *D. rerio*, *Bufo bufo*, *Gallus gallus*, *Rattus norvegicus*, and *H. sapiens*. The analysis showed high conservation among species from different taxa, with identities of 92.61% and 84.92% with *D. rerio* and *H. sapiens*, respectively (Supplementary Table S1).

**Experimental transcriptomic data of the head kidney of *S. salar***. Alignment of the transcriptome coding sequence with the reference sequence indicated a high degree of mRNA conservation, yielding a consensus sequence 100% matching the predicted in silico DNA model (Figure S1). Additionally, a single-nucleotide polymorphism (SNP) was identified at nucleotide 852 in Experiment 3. This variation in the coding sequence led to a silent mutation, as shown in the multiple alignment of amino acid sequences (Figure S1, bottom panel). These results of the *S. salar* head kidney transcriptomes indicated the gene’s transcription fidelity, with a high conservation level between the observed mRNA sequences and the predicted in silico DNA. Moreover, the SNP identified in the transcriptome analysis did not affect the predicted amino acid sequence of DDX41.

**Figure 2:**
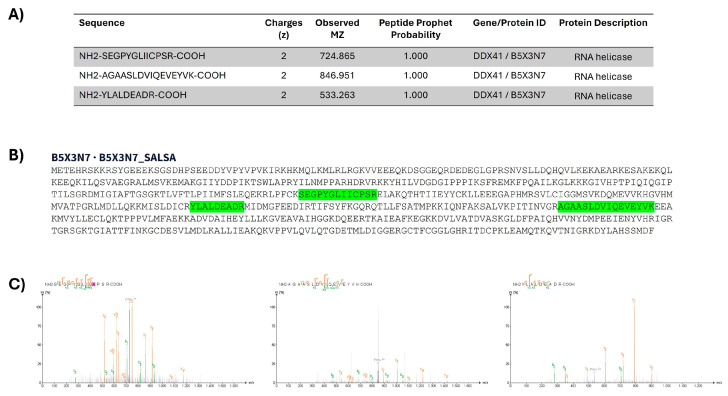
Proteomic identification of DDX41 in head kidney of *S. salar*. (**A**) Peptide identification indicates mass and probability of identification; (**B**) sequence and peptide identify are highlighted in green; (**C**) fragmentation spectra of each peptide identify; pink background highlight cysteine-alkylation.

**Proteomic identification of DDX41 in the head kidney of *S. salar***. Extracted proteins were prepared for a detailed bottom-up proteomics analysis via nLC-MS/MS, leading to the sequencing of a comprehensive total of 34,177 peptides and the identification of 5781 proteins. DDX41 from *S. salar* head kidney was successfully identified and sequenced. Among the proteins identified, the RNA helicase protein encoded by the *ddx41* gene was identified, characterized by three unique peptides with an exceptional statistical confidence of 99.99%, as detailed in the table presented in Figure 2A, where these peptides are highlighted in green (Figure 2B). To further validate the robustness of our protein sequencing and identification approach, we analyzed the fragmentation spectra of each peptide (Figure 2C). The precise assignment of “b” and “y” ions, as illustrated in Figure 2C, confirms the accuracy of our peptide-to-protein mapping process.

### 2.2. Prediction Origin, Phylogenetic, and Structural–Functional Analysis of ddx41 in S. salar

**Figure 3:**
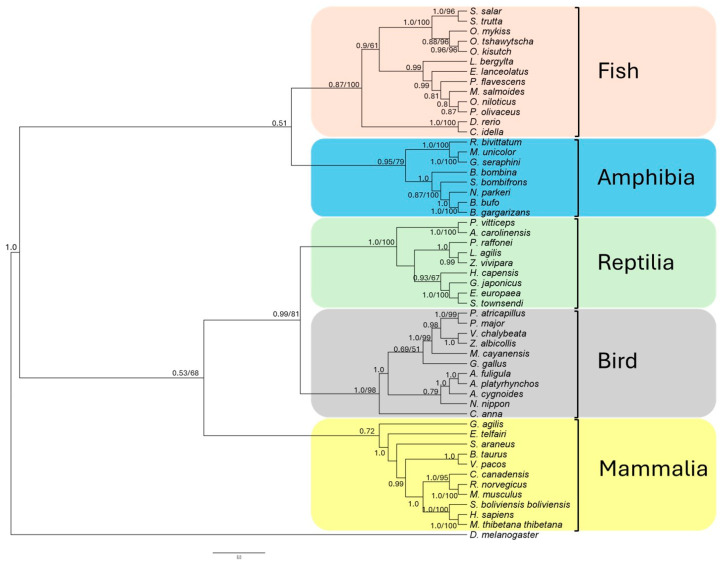
Phylogenetic analysis, conducted with 52 coding sequences from unrelated vertebrates, elucidates the evolutionary relationships of *ddx41* genes. Using MrBayes and the maximum parsimony method, the bootstrap consensus tree was inferred from 1000 replicates. Bayesian posterior probability and bootstrap support value are indicated at each node, representing common ancestors, as well as branching points where species or sequences diverged during evolution. *D. melanogaster* was included as an outgroup.

The evolutionary relationship and conservation of functional elements of the *ddx41* gene across various species, including *S. salar* and 51 other unrelated vertebrates, are presented in Table S1. Utilizing the nucleotide sequences of *ddx41* from humans and the predicted sequence from *S. salar*, along with sequences from a diverse range of vertebrates, we constructed a phylogenetic tree to elucidate the evolutionary trajectories of the *ddx41* genes. The phylogenetic analysis, supported by high Bayesian posterior probabilities (>0.9) and strong bootstrap values (>50%), revealed a monophyletic relationship among the taxa studied. Notably, the *ddx41* coding sequence was categorized into five distinct clusters corresponding to major vertebrate groups: Fish, Amphibia, Reptilia, Bird, and Mammalia (Figure 3). Moreover, the results showed that ddx41 sequence from *S. salar* and other salmonids (*Salmo trutta*, *Oncorhynchus mykiss*, *Oncorhynchus kisutch*, *Oncorhynchus tshawytscha*) formed a cohesive cluster, supported by 1.0 Bayesian probability and 98% likelihood, underscoring the robustness of the inferred evolutionary relationships. The comparative genomics analysis confirms the evolutionary conservation of the *ddx41* gene across vertebrates, with *S. salar* and closely related salmonids forming a distinct cluster. This highlights the gene evolutionary stability and the conservation of functional elements from human to the salmonid lineage, reinforcing the confidence in the gene evolutionary significance and functional conservation (Figure 3).

**Figure 4:**
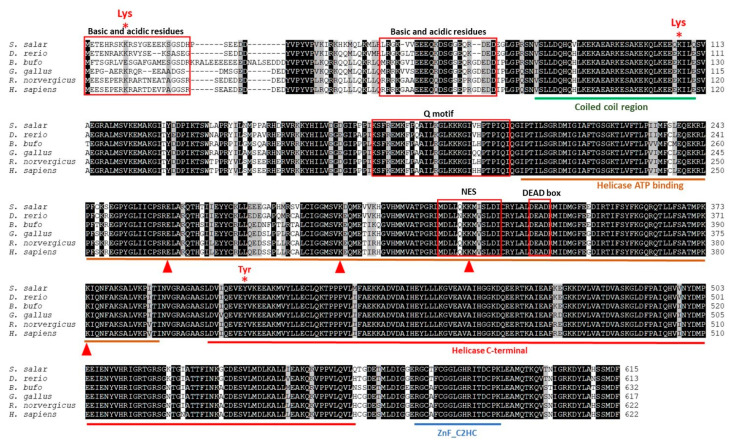
Multiple alignment of DDX41 deduced amino acid sequence from *S. salar*, *D. rerio*, *B. bufo*, *G. gallus*, *R. norvegicus,* and *H. sapiens*. Identical residues between organisms are highlighted in black color and amino acid with chemical similar characteristic are highlighted in grey color. Numbers on the right indicate the position of the last residue in the alignment relative to the complete amino acid sequence of each species. The conserver domain coiled coil, Q-motif, helicase ATP binding, helicase C-terminal, and ZnF_C2HC are indicated. The basic and acid residues, nuclear signal (NES), and DEAD box are boxed. Asterisk and triangles indicate the important residues in function of DDX41.

Furthermore, the alignment of the amino acid sequence showed that the coiled-coil (CC) region, Q-motif, helicase ATP binding, helicase C-terminal, and ZnF_C2HC domains are shared between these organisms (Figure 4). In the multiple sequence alignment, key residues previously identified in both *D. rerio* and *H. sapiens* were identified. These residues include Lys108, which is implicated in the negative regulation of DDX41 through proteasomal degradation after the activation of the STING pathway, and Tyr407, phosphorylated by BTK to activate DDX41 for DNA sensing. Also, specific residues essential for binding double-stranded DNA or cyclic dinucleotides, previously described in other organisms such as *D. rerio,* are denoted by triangles within the multiple alignments (Figure 4).

The measurement of the percent identity of each domain of DDX41 of *S. salar* with *D. rerio*, *B. bufo*, *G. gallus*, *R. norvegicus*, and *H. sapiens* showed a high amino acid similarity percent. The CC region shared 97.14% with all organisms analyzed. The Q-motif shared between 100 and 89.66% with these organisms, the helicase ATP binding shared between 97.03 and 94.05%, the helicase C-terminal shared between 99.38 and 98.13%, and ZnF_C2HC shared between 100 and 94.44% (Figure 4). Additionally, the Logo schemes of each motif and domine show high conservation between DDX41 of *S. salar* and organisms of different taxa (*D. rerio*, *B. bufo*, *G. gallus*, *R. norvegicus*, and *H. sapiens*) (Figure S2). Moreover, the scheme shows the high conservation of the DEAD box in the helicase ATP binding in the different organisms (Figure S2C).

The amino acid sequence of DDX41 was predicted using the EMBOSS Transeq tool in EMBL-EBI web service (https://www.ebi.ac.uk/jdispatcher/st/emboss_transeq, accessed on 16 April 2024) [30] and utilized for in silico protein characterization. The calculated molecular mass and theoretical isoelectric point were 69.12 kDa and 8.12, respectively. Subcellular localization prediction using Deep-Loc 2.0 tool and MuLocDeep indicated nuclear localization for DDX41 with probabilities of 0.8127 and 0.942, respectively.

**Figure 5:**
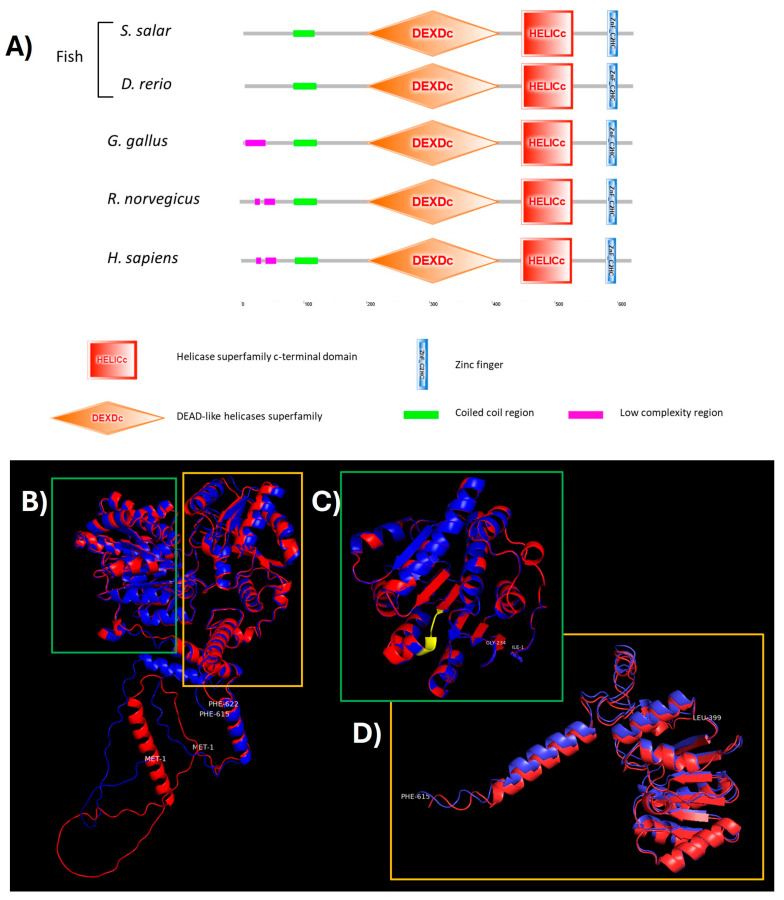
Comparative analysis of predicted DDX41 protein structures between vertebrates, including *S. salar*, alongside the crystal structure of human DDX41. (**A**) Principal domain in *S. salar*, *D. rerio*, *G. gallus*, *R. norvegicus*, and *H. sapiens*. Domains are color-coded and depicted in box forms. (**B**) Aligned 3D protein model of DDX41 with *H. sapiens* (red) and *S. salar* (blue), with residues labeled at the start and end of the protein chain. (**C**) Helicase C-terminal domain is highlighted in a green box. (**D**) Helicase ATP binding domain with DEADc domain is highlighted in a yellow box.

Subsequently, the identification of domains in the amino acid sequence of DDX41 of *S. salar* was performed with the SMART tool and compared with different vertebrates. The results showed the presence of canonical structural motifs for DDX41 proteins, particularly the helicase core motifs—an inherent characteristic of the DEAD-box helicase family. Moreover, the results showed the coiled-coil region in the amino-terminal, helicase superfamily c-terminal domain, and Zinc finger in the carboxyl-terminal. The domain’s architecture showed high conservation among vertebrates of different taxa (Figure 5A). These domains, defined by conserved sequence motifs, establish the ATP binding pocket crucial for ATP hydrolysis. Additionally, RecA-like domains (DEAD and HELICc domain), essential for nucleic acid binding, and the C-terminal domain further modulate helicase activity and nucleic acid interactions.

These conserved motifs, typically spanning the ATPase/helicase core domains, serve as molecular signatures essential for ATP binding, hydrolysis, and RNA duplex unwinding, essential for the recognition of foreign nucleic acids. The RecA-like domains play a fundamental role in nucleic acid binding, while the C-terminal domain further modulates helicase activity and nucleic acid interactions. Furthermore, these conserved boxes within DDX41 likely govern its oligomerization, fostering the assembly of higher-order complexes critical for signal transduction cascades upon pathogenic nucleic acid detection (Figure 5A).

Later, DDX41 protein models were generated according to the Materials and Methods section. The model displayed the complete DDX41 protein, highlighting the N-terminal methionine (Met1) and the C-terminal phenylalanine residues (Phe615 and Phe622) (Figure 5B). Further, a zoomed-in analysis revealed the helicase ATP binding domain with the DEADc motif accentuated in yellow (Figure 5C), and the helicase C-terminal domain (Figure 5D), both demonstrating high homology between the two species. The Local Distance Difference Test (LDDT) scores ranged from 74.8 to 79.3 for *H. sapiens* and from 76.4 to 79.2 for *S. salar*, indicating a significant structural similarity. Template modeling (TM) scores varied from 0.593 to 0.679 for *H. sapiens* and from 0.565 to 0.690 for *S. salar*. Moreover, the chosen models for each organism exhibited LDDT and TM scores of 74.8 and 0.678 for *H. sapiens* and 76.4 and 0.683 for *S. salar*, respectively.

The quality scores and predictions show better values for domains over complete protein according to every bioinformatic tool (Table 2). ERRAT revealed that the helicase C-terminal is the most conserved with the highest score, which is similar for both organisms. According to VERIFY 3D, helicase ATP binding for *H. sapiens* showed a score of over 80%, this one being the only one to pass the criteria of valid compatibility between models with amino acid sequences. The scarcity of crystallography samples of domains or DDX41 protein itself reduced the precision of prediction protein models. Finally, PROCHECK revealed the same results for both organisms, highlighting the best scores for helicase ATP binding, considering the use of template from PDB of the DDX41 domain from *H. sapiens*, and the worst result for complete protein, probably related to the N-terminal region before the coiled-coil region.

### 2.3. Expression Pattern of the ddx41 Gene across S. salar Tissues

In relation to the expression of the *ddx41* gene in *S. salar*, we initially evaluated the expression of mRNA from healthy specimens. The results showed the constitutive expression of the *ddx41* gene in an extensive array of tissues, including the spleen, heart, liver, gill, head kidney, posterior kidney, muscle, brain, and eyes. The expression level of the *ddx41* transcript was normalized against the lowest expression observed, which was consistently found in the liver (Figure S3). This normalization process underscores the gene’s active transcription into mRNA throughout various *S. salar* tissues, showing a significant uniform expression pattern.

### 2.4. Gene Expression of ddx41 and Inflammatory Factors in SHK-1 Cells Infected with Gram-Positive and Gram-Negative S. salar Pathogens

**Figure 6:**
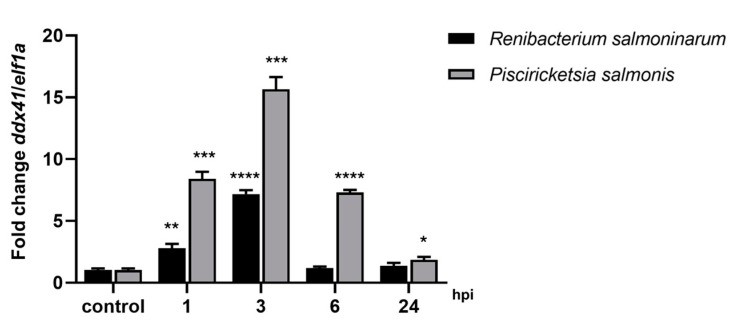
Immune response kinetics of *ddx41* gene expression were assessed from 0 (control) to 24 hpi in SHK1 cells following infections with *P. salmonis* and *R. salmoninarum*. qPCR quantified *ddx41* gene expression, normalized to elongation factor-1α (*elf1a*). Control: non-infected SHK-1 cells. Results, presented as mean ± SE, indicate significance with asterisks: (*) *p* < 0.05; (**) *p* < 0.01; (***) *p* < 0.001; (****) *p* < 0.0001.

In relation to the expression analysis of the *ddx41* gene, the in vitro assay using the *S. salar* SHK-1 cell line, we demonstrated the transcription and expression of the *ddx41* gene. Particularly, during the initial phase of intracellular bacterial infection, the *ddx41* gene exhibited overexpression at each instance of infection kinetics compared to the non-infected SHK-1 cell line as a control condition. The infection was carried out with *P. salmonis* (grey bar) and *R. salmoninarum* (black bar), as depicted in Figure 6. Examining gene expression levels during infection kinetics further indicated an increase in DDX41 expression after 1 h post-infection (hpi) for both pathogens, with a greater surge observed in *P. salmonis* infection. At 3 hpi, the highest expression levels were noted, particularly with infections from *R. salmoninarum* and *P. salmonis*. By 6 hpi, *ddx41* transcript levels decreased during infections with both pathogens, returning to basal levels at 24 hpi. Markedly, the infection kinetics for *R. salmoninarum* distinctly revealed basal levels of gene expression as early as 6 hpi.

**Figure 7:**
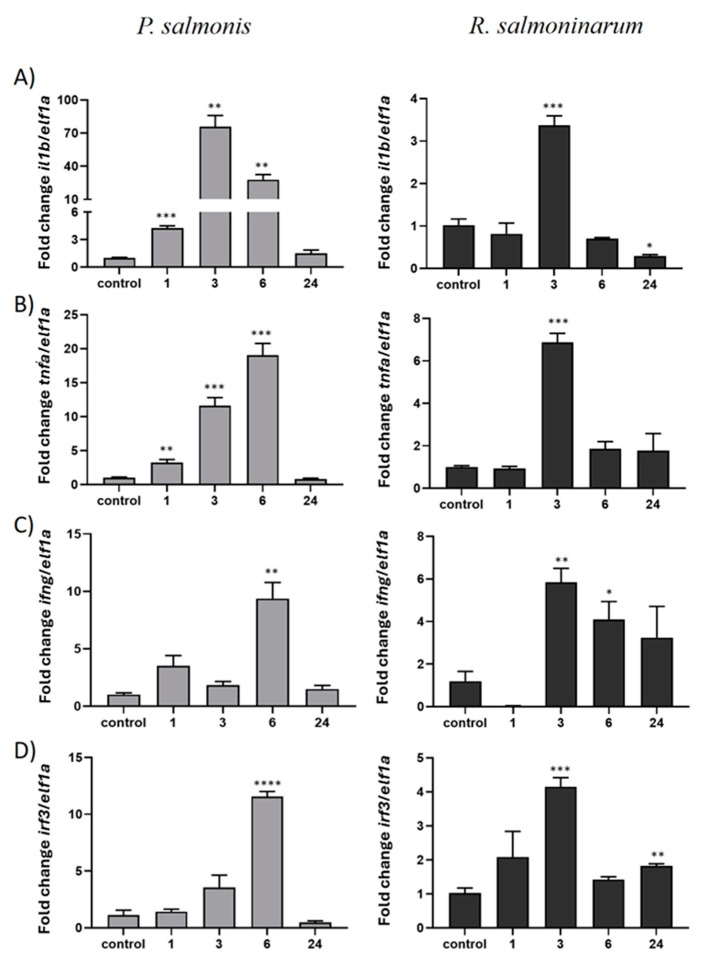
Innate immune response against intracellular pathogens in the SHK-1 cell line was assessed by measuring the gene expression levels of inflammatory genes *il1b* (**A**), *tnfa* (**B**), *ifng* (**C**), and *irf3* (**D**) during infection kinetics with *P. salmonis* and *R. salmoninarum* (0–24 hpi). Gene expression was analyzed using qPCR and normalized to the elongation factor-1a (*elf1a*) gene level. Control: non-infected SHK-1 cells. Data are presented as mean ± SE, with significance indicated by asterisks: (*) *p* < 0.05; (**) *p* < 0.01; (***) *p* < 0.001; (****) *p* < 0.0001.

Moreover, to gain insight into the active involvement of *ddx41* in the invasion process during infection with *P. salmonis* in SHK-1 cells, innate immune modulations were observed in genes encoding proinflammatory cytokines. The expression of each cytokine during infection kinetics was compared with the non-infected SHK1 cell line (control condition) (Figure 7). Overexpression of *il1b*, *tnfa*, and *ifng* was observed after 1 hpi. The highest level was observed at 3 hpi for *ilf1b*, while *tnfa* and *ifng* molecules showed increased expression levels later at 6 hpi, returning to basal expression levels at 24 hpi. Regarding the expression levels of the *irf3* signaling pathway gene, expression increased mostly at 6 hpi and decreased to basal levels at 24 hpi. Similarly, in the kinetics of infection with *R. salmoninarum* (Figure 7), higher expression levels of *il1b*, *tnfa*, *irf3*, and *ifng* were observed at 3 hpi, with a decrease in their expression levels at 6 and 24 hpi. The pattern of expression of genes related to immune response evaluated in SHK-1 cells was similar during the infection with *P. salmonis* and *R. salmoninarum* (Figure 7).

### 2.5. The Gene Expression of ddx41 and Immune Innate Genes in the Head Kidney of S. salar in Response to P. salmonis Challenge

As we describe, the SHK-1 cell line demonstrated an enhanced immune response to *P. salmonis*. Subsequently, an in vivo *ddx41* expression experiment was conducted in *S. salar* challenged with *P. salmonis*. Head kidney samples were collected from non-infected healthy fish as a control condition and infected the cohabitant fish at 0-, 7-, 14, 21, 28, 35, 42, and 49 days post-inoculation. 

**Figure 8:**
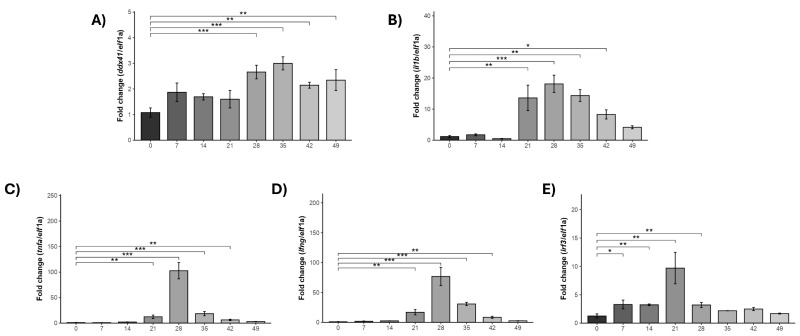
Evaluation of innate response in *S. salar* to natural infection with *P. salmonis*; the cohabitant (naive) group exhibited mortalities starting from 28 dpc, reaching 70% at 49 dpc. Head kidney samples were collected from *S. salar* between 0 and 49 days post-*P. salmonis* challenge. qPCR analysis measured gene expression of *ddx41* (**A**), *il1b* (**B**), *tnfa* (**C**), *ifng* (**D**), and *irf3* (**E**), normalized to elongation factor-1a (*elf1a*) levels. Control: non-challenged *S. salar.* Results are presented as mean ± SE, with significance indicated by asterisks: (*) *p* < 0.05; (**) *p* < 0.01; (***) *p* < 0.001.

The results showed that in the following tank, the Trojan fish group, IP-injected with *P. salmonis*, experienced fish mortalities beginning at 12 days post-challenge (dpc), resulting in 100% mortality by 23 dpc. Conversely, the naive fish cohabitant group in our study displayed mortalities starting at 28 dpc, reaching 100% mortality by 49 dpc (Figure S4). Then, an evaluation of gene expression in this tissue revealed a significant upregulation of *ddx41* and *il1b* starting from day 28 post-challenge (Figure 8A,B). The *ddx41* gene expression levels remained over 2-fold until 42 dpi. Additionally, genes encoding proinflammatory cytokines, including *IL-1β*, *TNFα*, and *IFNγ*, displayed a significant surge in expression in challenged *S. salar* (Figure 8B–D). At 28 dpc, *il1b*, *tnfa*, and *ifng* exhibited remarkable overexpression, peaking at 20-fold, 90-fold, and 70-fold higher than the control, respectively, with a denoted steady decline until 42 and 49 dpc (Figure 8C). However, the increased expression of these three genes was observed to start at 21 dpc. Correspondingly, the expression levels of the *irf3* gene reached their highest point at 21 dpc before returning to basal levels between 35 and 49 dpc (Figure 8E). The increased expression of *ddx41* at 28 dpc coincided with the detection of *P. salmonis* in the tissue (Figure S5), emphasizing its role in innate immunity against intracellular pathogens during the cohabitation challenge. Cytokines *il1b*, *tnfa*, and *ifng* showed substantial overexpression at 28 dpc, reinforcing a coordinated defense. This parallel response highlights the involvement of *ddx41* in the innate immune response at the organ level, specifically in the head kidney, a critical immune organ in fish. It is worth mentioning that the gene normalizer showed uniform behavior across the samples, which reinforces the detected differences in gene expression (Figure S6).

## 3. Discussion

The entry of a pathogen into the host cell initiates complex interactions between several pathogen-derived molecules and host sensors [31,32]. Recognizing the presence of invading pathogens is crucial for developing an effective innate immune response. Both fish and mammalian cells express different classes of pattern recognition receptors (PRRs) in subcellular compartments [33,34], which alert the cell to any signs of infection and activate several signaling pathways, resulting in a complex interaction [15]. Recent advances in identifying different types of PRRs in teleost fish have revealed several intracellular sensors for the recognition of viral and bacterial PAMPs [21,24,25,34,35]. Comparative immunology necessitates an understanding of shared immune factors across species [36]. These specific interleukin distinctions underscore the divergence in immune regulatory pathways between *S. salar* and *H. sapiens*. Therefore, studying the existence of a gene as an immunological player from humans to *S. salar* is essential, revealing species-specific adaptations in immune signaling functions, especially in *il2*, *il4*, *il6*, and *il10* [37,38,39,40].

The *ddx41* gene, known extensively in mammals as an intracellular sensor for pathogen infection [15], lacks substantial information about its existence within the genome and translation in *S. salar*. This study emphasizes the existence of the orthologous *ddx41* gene in the genome of *S. salar*, marking the first documentation of its presence in this species as a newly discovered contributor to the immune response of *S. salar* against intracellular pathogens. DDX41 serves as a primary immune sensor in *S. salar*, akin to its functions in other vertebrates. The results of in silico analysis, utilizing BLAST, identified a homologous *ddx41* gene in *S. salar*, revealing a singular copy on chromosome 5 with a complex gene structure; the presence of a single *ddx41* ortholog in the *S. salar* genome, with a gene structure comprising 17 exons and 16 introns over a 71.3 megabase region underscoring the gene’s potential complexity and regulatory capacity. This finding underscores the evolutionary conservation and potential regulatory complexity of the *ddx41* gene, suggesting its significant role in immune responses across species. Also, the genomic analysis showed 78.68% nucleotide identity and 83.92% protein between *S. salar* DDX41 and its human counterpart, highlighting a close evolutionary relationship and suggesting the conservation of functional aspects of the *ddx41* gene. This high degree of conservation, especially at the protein level, indicates the gene’s crucial role in the innate immune system, potentially in recognizing cytosolic DNA and initiating antiviral responses [17]. The comparative analysis with other species like *R. norvegicus* and *G. gallus* enriches our understanding of *ddx41*’s evolutionary trajectory, emphasizing its fundamental role across vertebrates.

The in silico identification of the *ddx41* gene in *S. salar* provokes critical inquiry into its transcriptional activity, moving from potential genomic roles to actual mRNA expression. This step is essential to understand the gene’s true function and significance in the organism’s cellular processes. Utilizing public transcriptomic data, our investigation into the head kidney transcriptomes of *S. salar* has demonstrated that the *ddx41* gene is transcribed into mRNA with a high degree of conservation to the previous predicted in silico *ddx41* gene sequence of the *S. salar* genome. Moreover, the results allowed the identification of a silent SNP, maintaining the gene evolutionary stability, suggesting its critical role in immune responses in diverse organism [15,17]. Additionally, the proteomic analysis of the head kidney from *S. salar* confirmed the bioinformatic analysis, demonstrating that the mRNA is translated into protein. This represents the first combined genomic and proteomic analysis providing evidence of the existence of the orthologous DDX41 gene in *S. salar*, which shows a high similarity to its human counterpart. Our findings enhance the understanding of salmonid immunology, potentially guiding future strategies to improve disease resistance in aquaculture.

The phylogenetic and structural analysis confirms the substantial conservation of *ddx41* across vertebrates, highlighting its key role in the innate immune system [15]. Extensively studied in humans, mice, and more recently in teleost like *D. rerio* [21], DDX41 exhibits a distinct cluster for fish nucleotide sequences, a classification observed in other fish species but not in salmonids [24]. Comparative phylogenetic analysis of protein sequences, maintaining 83.92% identity between *H. sapiens* and *S. salar* in silico gene, provides intriguing high evolutionary insights. This approach aids in discerning conserved regions crucial for protein functionality, offering a profound understanding of evolutionary dynamics and shared functionalities or adaptations [21]. Phylogenetic analysis uncovers the complex evolutionary paths of proteins across different species. Notably, the consistent conservation of gene structures when compared to humans underscores significant evolutionary stability and highlights the functional significance of orthologous genes. This conservation across distant eukaryotes, such as *S. salar* and humans, signifies profound evolutionary preservation [41].

The exploration of DDX41 protein domains in *S. salar* unveils structural motifs crucial to study its immunological function. Within DDX41 protein analysis of *S. salar*, the conserved motifs primarily encompass distinct structural–functional domains, notably the helicase core motifs characteristic of the DEAD-box helicase family. Our study across diverse species, including *S. salar*, *D. rerio*, and *H. sapiens*, reveals significant amino acid sequence conservation in the DDX41 protein, highlighting its evolutionary importance [30]. The coiled-coil region and other domains like the Q-motif and helicase ATP binding show similarities ranging from 89.66% to 100%, emphasizing DDX41’s conserved roles across taxa [42]. Utilizing the WebLogo tool, we observed high conservation in DDX41 motifs, particularly the DEAD box within the helicase ATP binding domain, suggesting its critical function in RNA metabolism and innate immunity. In silico analysis confirmed DDX41 nuclear localization and detailed its structure, with high Local Distance Difference Test (LDDT) and template modeling (TM) scores indicating significant structural similarity across species [43,44]. Despite challenges in model prediction due to limited crystallography samples, bioinformatics tools like ERRAT and VERIFY 3D provided insights into domain conservation and sequence compatibility, especially for the helicase ATP binding domain [43,44].

These domains, typified by conserved sequence motifs such as Walker A and Walker B motifs, form the ATP binding pocket essential for ATP hydrolysis [45], a pivotal step in RNA unwinding. Moreover, the structural architecture of DDX41 features additional conserved functional domains, including the RecA-like domains and the helicase C-terminal domain, key for RNA duplex recognition and unwinding activity [17]. The RecA-like domains play a fundamental role in nucleic acid binding, while the C-terminal domain further modulates helicase activity and nucleic acid interactions [46]. The interplay between these conserved functional domains composes DDX41’s ability to discern and bind foreign RNA species. This interaction triggers conformational changes essential for downstream signaling, enabling the assembly of higher-order protein complexes crucial for immune signaling cascades. The results of amino acid characterization of *S. salar* DDX41 showed high identity with *D. rerio* and contained DEADc, HELIc, and Zinc-finger conserved domains previously described in zebrafish, humans, and other vertebrates [21]. Moreover, the amino acid characterization by multiple alignments and 3D structure prediction showed highly conserved sequence domains among organisms, including mammals, birds, and amphibians (Figure 2, Figure 5, Figure 6 and Figure 7). The high similarity in amino acid sequence and functional domain between DDX41 of *S. salar* and orthologous in other vertebrates (Teleostei, Amphibia, Birds, and Mammalia) suggests the functional conservation in the innate immunity response during vertebrate evolution [21].

Genomic findings suggest that a structural protein within the helicase superfamily 2 (SF2) has maintained its essential intracellular function. Notably, this protein holds a unique distinction as it can recognize various types of ligands, including double-stranded DNA, RNA, and cyclic dinucleotides (CDNs) [45]. This cyclic di-nucleotide, synthesized from two GTP molecules by diguanylate cyclase, serves as a bacterial secondary messenger for signal transduction [47]. Despite pathogen-associated molecular patterns (PAMPs) being identified from *P. salmonis* and *R. salmoninarum*, investigations into secondary messengers in these bacteria remain scarce. Various bacterial pathogens, including *Staphylococcus*, *Streptococcus*, *Pseudomonas*, *Yersinia*, *Listeria*, and *Mycobacteria*, employ these secondary messengers to modulate host cell functions [9]. Moreover, Parvatiyar et al. [11] demonstrated that the helicase DDX41 recognizes bacterial cyclic dinucleotides, particularly c-di-GMP, activating the inflammasome pathway. A similar mechanism might underlie the response to *P. salmonis* and *R. salmoninarum* infections in *S. salar*, elucidating the activation of DDX41 and subsequent immune reactions. Upon detecting these nucleic acids, DDX41 triggers and activates an innate immune response. This versatility in ligand recognition is important in the context of intracellular pathogen recognition. Crystal structure analysis of the DDX41 HELICc domain uncovered structural parallels with HELICc domains present in other DEAD box proteins [48]. These findings strongly indicate that the identified *ddx41* gene in *S. salar* maintains a highly conserved structure, safeguarding fundamental functional domains crucial for DDX41’s sentinel role. Comparative amino acid study underscores the significance of residues, shedding light on the function of conserved domains and regulatory amino acids. Additionally, elucidating the function of conserved domains reported in humans solidifies the understanding of their importance in the DDX41 protein in *S. salar*. Notably, previous research has elaborated on the functional relevance of these conserved domains, providing crucial insights into their roles within the DDX41 protein structure [11,17,45].

Interestingly, as a regulatory mechanism following the immune response, DDX41 undergoes ubiquitination by Trim21 at Lys9 and Lys115 sites, ultimately resulting in proteasomal degradation [22]. The extensive analysis of this mammalian model encourages deeper investigation within fish, leveraging the insights gained from this *S. salar* study. Significantly, the unique clustering of *ddx41* gene sequences in the salmonid lineage is characterized by a differentiated amino terminus that does not conserve Lys9. However, the conservation of Lys115, a crucial regulatory residue, remains consistent across all studied species [22]. The evolutionary conservation of structural function in DDX41 motifs highlights the significance of this gene as a pivotal player in immune responses across diverse organisms, with a particular emphasis on the extensively studied DDX41 protein in humans [14,15,21]. In general, pathogens such as viruses and bacteria release dsDNA or c-di-GMP into cells by activating the intracellular sensor DDX41, which is phosphorylated by BTK kinase at the Y414 site. Activated DDX41 can sense the DEAD domain containing PAMP and then activate STING, which then translocates from the endoplasmic reticulum (ER) to the Golgi apparatus and interacts with TBK1 [49]. The activation of TBK1 and subsequent phosphorylation and nuclear translocation of IRF3 ultimately lead to the expression of type I interferons. Such a mechanism extensively studied in mammalian models should likewise be further studied in fish, building on the groundwork from this *S. salar* study.

Therefore, in this cell infection model, we further determined the functional gene expression levels of the DDX41 pathway, including the activation of IRF3 and downstream immune signaling pathways of *il1b* and *tnfa* genes following bacterial infection, which underscore the DDX41 gene’s central role in mediating immune responses in *S. salar*. However, the preservation of these conserved motifs across species emphasizes their evolutionary importance in immune surveillance.

The consistent mRNA transcription across diverse tissues implies a stable baseline of DDX41 activity in *S. salar*. Normalizing its transcript to the least observed expression underscores its ubiquitous presence, suggesting regulatory mechanisms maintain this expression level. The widespread presence of *ddx41* across healthy tissues suggests its involvement in crucial roles spanning various physiological functions, potentially extending beyond its known immune-related functions such as nucleic acid metabolism. Its active transcription into mRNA across a broad spectrum of tissues highlights its importance in the biology of *S. salar*, warranting further investigation into its specific functions and regulatory mechanisms. Notably, prior studies on *ddx41* expression in fish species have been limited [25], but the presence of *ddx41* has been limited and only documented in fish species like *D. rerio*, *P. olivaceus*, *E. coioides*, *O. niloticus* and *S. chuatsi*. In *E. coioides*, studies by Liu et al. noted prevalent expression in the gills, brain, and liver, suggesting diverse physiological and immunological functions in zebrafish [24]. DDX41, as a member of the DEAD box helicase family, has been shown to play a role in RNA metabolism, potentially contributing to processes such as RNA unwinding, RNA binding, or RNA–protein interactions. 

Functional studies, including knockout experiments, are crucial to unravel the specific biological functions governed by DDX41 in *S. salar*. Results published on *D. rerio*’s DDX41-mediated signaling pathways demonstrate its vital role in innate antibacterial immunity. The knockdown of DDX41 significantly reduced zebrafish survival upon *A. hydrophilia* or *E. tarda* infection, highlighting its importance in combating bacterial infections [20]. Similarly, in humans, DDX41 exhibits potential relevance in detecting and combating RNA viruses like influenza A virus and DNA viruses such as herpes simplex virus (HSV) and human cytomegalovirus (HCMV) [50]. Its involvement in recognizing viral RNA intermediates and DNA viral intermediates underscores its pivotal role in initiating immune activation against a spectrum of pathogens including intracellular bacteria.

In our in vitro assay, the *S. salar* SHK-1 cell line demonstrated robust transcriptional activity and expression of the *ddx41* gene, notably heightened at 3 h post-infection during the initial phase of intracellular bacterial infection. As an intracellular sensor, DDX41 promptly responds to pathogenic bacteria, triggering interferon expression. Its marked upregulation following challenges with *P. salmonis* and *R. salmoninarum* underscores its role as a swift intracellular sentinel against bacterial incursions. The kinetics of *ddx41* expression, peaking early post-infection and subsequently declining, suggest its involvement in instigating and modulating the early immune responses against these intracellular pathogens. This surge in *ddx41* expression correlates with heightened proinflammatory cytokines (IL-1β, TNFα, and IFNγ) and activation of the IRF3 signaling pathway, highlighting DDX41’s central role in activating a robust immune reaction in *S. salar* upon encountering bacterial challenges.

DDX41’s role in sensing nucleic acids is key in enabling the body to mount effective innate immune responses against a variety of pathogens, contributing significantly to the body’s defense mechanisms. It is noteworthy that DDX41’s function spans the recognition of bacterial DNA, RNA, D-cyclic AMP, and GMP, encompassing pathogens like *L. monocytogenes* and *Francisella tularensis* [11,51,52,53]. Upon bacterial infection, these pathogens release their genetic material into the host cell, with DDX41 acting as a key sensor, initiating immune signaling cascades, and prompting innate immune responses to clear the infection. This essential role in identifying diverse bacterial nucleic acids underlines DDX41’s significance in host defense mechanisms against intracellular bacterial infections [52]. Functionally, DDX41 serves as an intracellular DNA sensor in myeloid dendritic cells, modulating critical factors like TANK binding kinase 1 (TBK1), NF-κB, and IRF3, fundamental in type I IFN expression [45,54,55]. Operating through the stimulator of interferon genes (STING)-TBK1-IRF3 pathway, DDX41 interacts directly with DNA and STING, crucially mediated by its DEAD domain and Walker motifs A and B [17,56,57]. Some bacteria release cyclic di-GMP or cyclic di-AMP, activating host type I interferon immune responses [22]. DDX41 interacts specifically with c-di-GMP, influencing immune reactions; inhibiting DDX41 results in STING-TBK1-IRF3 pathway activation, suggesting a three-molecule binding mechanism: c-di-GMP detection enhances DDX41-STING interaction, strengthening STING c-di-GMP binding and leading to interferon response activation [11,20]. This comprehensive understanding emphasizes DDX41’s fundamental role in pathogen recognition and immune response initiation against intracellular bacteria. The dynamic activation gene expression levels of *ddx41* at different infection stages in the SHK-1 cell line reveal the intricate regulatory mechanisms governing the cellular response during this critical biological event.

Understanding the modulation of *ddx41* gene expression during various stages of Piscirickettsiosis in *S. salar* provides deep insights into immune dynamics and host–pathogen interactions. As an intracellular sensor, DDX41 triggers IRF3 and interferon responses, like other known intracellular sensors, rapidly activating upon the detection of pathogenic bacteria. This pronounced upregulation following challenges with *P. salmonis* highlights its role as an efficient sentinel, essential for early immune detection and response. Further insights come from assessing the cohabitation challenge, where the mortality patterns between cohabitation and intraperitoneal (IP) groups underscore the complex interplay affecting disease outcomes. The upregulation of the *ddx41* gene in the head kidney at 28 days post-challenge coincides with the PCR detection of *P. salmonis* and the onset of fish mortality, underscoring its significant role in initiating immune responses. Consistent expression patterns of cytokines such as IL-1β, TNFα, IRF3, and IFNγ during in vivo challenges reinforce DDX41’s central role in the innate immune defense within the head kidney. These observations across both in vitro and in vivo studies emphasize DDX41’s rapid activation upon encountering *P. salmonis*, marking it as a critical early immune sensor and a potential target for enhancing disease resistance in aquaculture.

Both in vitro and in vivo results highlight DDX41’s rapid upregulation upon *P. salmonis* detection, underscoring its role as an early immune sensor. In SHK-1 cells, *ddx41* expression peaks at 3 h post-infection, demonstrating its quick response to intracellular pathogens. Similarly, in the head kidney of *S. salar*, *ddx41* is swiftly upregulated following pathogen detection by PCR at 28 days post-challenge. This consistent early response across different models emphasizes DDX41’s crucial role in initiating defense mechanisms against infections. These findings suggest that targeting DDX41 could enhance disease resistance in salmon, providing a strategic focus for aquaculture health management. This consistent expression pattern, particularly in the head kidney, a primary immune organ, underscores its potential as a biomarker for intracellular infections in aquaculture.

Increased expression of DDX41 in the head kidney of *S. salar* following infection with intracellular bacteria suggests its potential as a diagnostic marker for such infections in salmonids and highlights its role in the innate immune response at this crucial immune organ in fish. Similarly, recent research by Feng-Ying et al. demonstrated overexpression of the *irf3* gene in Tilapia macrophages, underscoring the activation of the innate immune system through IRF3 and reinforcing the critical role of DDX41 in orchestrating a robust immune response in *S. salar* upon bacterial encounter [35]. qPCR techniques offer valuable insights into the immune response to pathogens [11]. Current efforts are focused on refining methodologies to accurately determine DDX41’s regulation of gene expression in innate immune responses, with future studies planned in which the SHK-1 cell line will be used to reduce the use of live fish and achieve a reduction in research immunology.

## 4. Materials and Methods

### 4.1. Bioinformatic Study: Identification, Structural Analysis of Salmo salar ddx41 Gene, and Phylogenetics

#### 4.1.1. Data Retrieval and Sequence Alignment

The identification of the homologous *ddx41* gene in *S. salar* was carried out employing human *ddx41* sequences as analogous templates. The analysis was conducted using BLAST (Basic Local Alignment Search Tool) (https://blast.ncbi.nlm.nih.gov/Blast.cgi, accessed on 3 August 2023) [58]. A comparative analysis of *ddx41* from different vertebrates was performed through BLASTN and BLASTP searches [48] against *ddx41* of *H. sapiens* (AK222598.1/BAD96318.1), retrieving query usage, E-value, and identity percentage.

#### 4.1.2. mRNA Sequence Analysis of *S. salar* Head Kidney Transcriptomes

We aligned these sequences with the reference *S. salar* mRNA (NM_001140327.1) using Clustal (v. 2.0) [59], incorporating data from various submission years. The experimental details are as follows: Experiment 1 (2021, GIYK01062020.1), Experiment 2 (2017, GGAQ01023159.1), Experiment 3 (2016, GEGX01092009.1), and Experiment 4 (2014, GBRB01046301.1), covering the full gene length. For the visualization of both nucleotide and amino acid sequences, we employed the Jalview tool (v. 2.11.3.1) [60]. Our analysis of the *ddx41* gene in *S. salar* head kidney transcriptomes demonstrated a consistent transcriptional pattern across different years, highlighting the gene’s evolutionary conservation.

#### 4.1.3. Model Selection and Phylogenetic Analysis

The coding sequence of the human ddx41 gene, along with predicted sequences from *S. salar* and 50 vertebrates across various taxa (Table S1), were retrieved from the NCBI database on 10 November 2023. Moreover, the *ddx41* coding sequence of *Drosophila melanogaster* (accession number NM_079496.4) was included as an outgroup in the phylogenetic analysis. The best-fit DNA substitution model was determined using MrModeltest (v. 2.4) [61], which identified the generalized-time-reversible (GTR) model with gamma distribution (G) and invariant sites (I), or GTR+G+I, as the most suitable. Phylogenetic trees were constructed using the maximum parsimony method with MEGA X (v. 10.1.8), incorporating 1000 bootstrap replicates [62], and Bayesian inference via MrBayes (v. 3.2.6) [63], employing 1 million generations and a 25% burn-in period. Both analyses utilized the GTR+G+I model. The resulting phylogenetic trees were visualized using FigTree (v. 1.4.4) [64]. This comprehensive approach underscores the gene’s evolutionary significance and provides a solid foundation for further comparative genomic studies.

#### 4.1.4. Structural Analysis and Protein Models

The amino acid sequencing was predicted with the EMBOSS Transeq tool [30] and the isoelectric point and relative molecular weight of DDX41 were calculated with the ExPASy server (https://web.expasy.org/protparam/, accessed on 03 August 2023). The subcellular localization prediction was developed using the Deep-Loc 2.0 tool (https://services.healthtech.dtu.dk/services/DeepLoc-2.0/, accessed on 03 August 2023) and MuLocDeep (https://www.mu-loc.org/, accessed on 03 August 2023). Multiple alignments of the amino acid sequence and visualization were performed with BioEdit software (v. 7.7.1.0). Moreover, the consensus sequences of each motif and domain were represented using the WebLogo tool (v. 2.8.2) [65]. Later, the prediction of the domain was developed with the SMART tool (v. 9.0) [66]. Protein models were generated with Alphafold2 (https://alphafold.ebi.ac.uk/, accessed on 25 March 2024) [67,68,69,70,71] for the complete protein and helicase C-terminal and Modeller (v. 10.4) [72] for the ATP binding helicase domain with 5GVR as annealing [17]. Subsequently, verifications and statistics were calculated with ERRAT (https://saves.mbi.ucla.edu/, accessed on 25 March 2024) [44] for non-bonded interactions between different atom types, VERIFY 3D (https://saves.mbi.ucla.edu/, accessed on 25 March 2024) for the compatibility of 3D atomic models with 1D amino acid sequences [73,74], and PROCHECK (https://saves.mbi.ucla.edu/, accessed on 25 March 2024)for assessing the stereochemical quality of protein structure by residue-by-residue geometry [43]. Finally, the models generated were visualized using PyMOL (v. 2.5.5) [75].

### 4.2. Experimental Animals

Sample Collection: Tissue samples from 10 healthy *S. salar* (mean weight 100 ± 10 g) were obtained from the Salmon Clinical Trials Facility, Institute of Animal Pathology, Faculty of Veterinary Medicine, Universidad Austral de Chile. Prior to experimentation, fish were acclimatized in fiber-reinforced tanks (1000 L) at 16 °C with filtered seawater + UV, with a photoperiod of 12 h light and 12 h dark. They were fed commercial pellets at a rate of approximately 1% *w*/*w* of their biomass. Fish were anesthetized and sacrificed, and tissues (anterior and posterior kidney, spleen, liver, heart, muscle, brain, eye, and gills) were collected for RNA extraction, stored in RNAlater (Life Technologies, Carlsbad, CA, USA), and kept at −80 °C until use.

Ethical Approval: all experimental protocols were approved by the Bioethics Committee of the Universidad Austral de Chile (Number 440/2021).

### 4.3. Proteomic Identification and Characterization of DDX41 from S. salar Head Kidney

#### 4.3.1. Protein Extraction for nLC-MS/MS

The *S. salar* head kidney was subjected to protein extraction by incubating for 10 min in lysis buffer (8M urea, 1% *w*/*v* SDS, 2% *w*/*v* Deoxycholate in 25 mM ammonium bicarbonate pH 8) (Merck, Darmstadt, Germany) and then subjected to disruption of the tissue by ultrasound (1 min with 3 pulses of 9 s at 40% intensity). Finally, the samples were centrifuged at 10,000× *g* for 10 min; the pellet was discarded and the supernatant was stored at −80 °C.

#### 4.3.2. Protein Extraction and Digestion for nLC-MS/MS

The proteins of each sample were subjected to precipitation using 5:1 *v*/*v* cold acetone 100% and incubated overnight at −20 °C, then centrifuged at 15,000× *g* for 10 min; the supernatant was discarded, and the pellet was washed three times with acetone at 90% *v*/*v*, dried in a rotary concentrator at 4 °C, and finally resuspended in 8M urea with 25 mM ammonium bicarbonate (NH_4_HCO_3_, pH 8.0) (Merck, Darmstadt, Germany). The concentration of proteins in each sample was quantified with a Qubit protein assay (Thermo Fisher Scientific, Waltham, MA, USA). Later, 100 µg of proteins of each sample was reduced with 20 mM dithiothreitol for one hour, alkylated with 20 mM iodoacetamide (Merck, Darmstadt, Germany) in the dark for one hour, diluted ten times with 25 mM ammonium bicarbonate pH 8.0 (Merck, Darmstadt, Germany), and digested with trypsin/LyC (Promega, Madison, WI, USA) in a 1:50 ratio overnight at 37 °C. Peptides were cleaned using Pierce C-18 Spin Columns (Thermo Scientific, Waltham, MA, USA) according to the manufacturer’s instructions, and the eluted peptides were dried using a rotary concentrator at 4 °C, resuspended in 2% *v*/*v* acetonitrile with 0.1% *v*/*v* of formic acid (Merck, Darmstadt, Germany), and quantified using a Direct Detect Spectrometer (Merck Millipore, Burlington, MA, USA).

#### 4.3.3. Liquid Chromatography

A nanoElute^®^ liquid chromatography system (Bruker Daltonics, Billerica, MA, USA) was used, and peptides (200 ng of digest) were separated within 44 min at a flow rate of 400 nL/min on a reversed-phase column Bruker fifteen (15 cm × 150 µm i.d. C18 1.9 µm) (Bruker Daltonics, Bremen, Germany) at 50 °C. Mobile phases A and B were water and acetonitrile with 0.1% *v*/*v* formic acid, respectively. The B percentage was linearly increased from 2 to 35% within 38 min, followed by an increase to 85% B within 2 min, followed by a washing step at 95% B and re-equilibration.

#### 4.3.4. The timsTOF Pro Mass Spectrometer

All samples were analyzed, and three biological replicates for condition on a hybrid Trapped Ion Mobility Spectrometry (TIMS) quadrupole time-of-flight mass spectrometer (MS) (timsTOF Pro) (Bruker Daltonics, Billerica, MA, USA) via a CaptiveSpray nano-electrospray ion source. The MS was operated in data-dependent mode for the ion mobility-enhanced spectral library generation. We set the accumulation and ramp time to 100 ms each and recorded mass spectra in the range of *m*/*z* 100–1700 in positive electrospray mode. The ion mobility was scanned from 0.85 to 1.3 Vs/cm^2^. The overall acquisition cycle of 0.53 s comprised one full TIMS-MS scan and four parallel accumulation–serial fragmentation (PASEF) MS/MS scans.

#### 4.3.5. Database Searching

Tandem mass spectra were extracted by TIMS Control version 4.1.13. Charge state deconvolution and de-isotoping were not performed. All MS/MS samples were analyzed using Fragpipe (v 2.0). For the protein search was set up to search the UniProt_SwissProt Salmo Salar database (82,312 entries) assuming the digestion enzyme trypsin. MSFragger (v. 3.8) was searched with a fragment ion mass tolerance of 10 ppm and a parent ion tolerance of 40 ppm. Carbamidomethyl of cysteine was specified in MSFragger as a fixed modification. Deamidated asparagine and glutamine, oxidation of methionine, acetyl of the n-terminus, and carbamyl of lysine and the N-terminus were specified in MSFragger as variable modifications. Statistical validation of protein identification was performed using peptide prophet, a closed search, a false discovery rate of 0.01, and a decoy database.

### 4.4. Infection Assay in SHK1 Cell Line with Pathogenic Bacteria

Infection Kinetic Assays: The Salmon Head Kidney-1 (SHK-1) cell line representing *S. salar* macrophages was used for infection kinetic assays [76,77,78]. Cells were infected with the *P. salmonis* strain AUS-005 and *R. salmoninarum* strain SF2022. Details of the culture conditions, infection procedures, and sampling time points were followed as outlined. SHK-1 cells were routinely maintained at 18 °C in Leibovitz L-15 medium (Gibco, Carlsbad, CA, USA) supplemented with 10% *v*/*v* fetal bovine serum (FBS) (HyClone, South Logan, UT, USA). Cells were grown to 90% confluency in 25 cm^2^ bottles. Twenty-four hours prior to infection, the cells were incubated with L-15 medium supplemented with 2% *v*/*v* FBS, incubated at 18 °C.

For in vitro assays, the *P. salmonis* strain AUS-005 was grown under standard conditions in Austral-SRS broth medium for 5 days at 18 °C and 180 rpm shaking [79,80]. *R. salmoninarum* strain SF2022 was cultured on KDM-2 medium [81]. The morphology and identity of these bacteria were confirmed by Gram staining, polymerase chain reaction (PCR), and an antibody immunofluorescence test (IFAT) (IFAT, SRS-Bios Chile; BKD FluoroTest Ango), following the instruction manual.

Infection kinetics in SHK-1 cells was induced by inoculating SHK-1 monolayer cells with 3 mL of Leibovitz L-15 medium (Gibco, Carlsbad, CA, USA) containing a culture of *R. salmoninarum* or *P. salmonis* at a multiplicity of infection MOI: 10 cfu/cell. The cells were incubated for 1 h at 18 °C as an infection procedure after the cells were washed with sterilized 1× PBS buffer to remove excess non-internalized bacteria. Fresh Leibovitz L-15 medium (Gibco, Carlsbad, CA, USA) was added, and sampling occurred at 1, 3, 6, and 24 h post-infection (hpi), with two biological replicates for each infection time. Cells were collected at each time point for subsequent RNA extraction, cDNA synthesis, and gene expression evaluation. Furthermore, cell culture bottles with non-infected SHK-1 cell lines were used as a control condition for each infection assay.

### 4.5. Cohabitation Challenge with P. salmonis

Experimental Setup: The in vivo component was conducted with *P. salmonis* to align with the reduction in experimental animal use principles. Atlantic salmon smolts (*S. salar*), weighing 140–170 g, were subjected to a cohabitation challenge using a *P. salmonis* EM-90-like strain, adhering to protocols reported by Herrera et al. [76]. The challenge involved an infectious dose of 10^6^ CFU per fish, administered via an intraperitoneal injection of 0.1 mL of bacterial inoculum. 

The experimental setup consisted of two 500 L tanks of seawater that was filtered, UV-treated, and maintained at 14 °C and a salinity of 32 ppt. Each tank housed 120 fish: 80 Trojan fish, marked by the removal of the upper lobe of the caudal fin and injected with the bacterial inoculum, and 40 uninfected cohabitants, maintaining a density of less than 40 kg/m^3^. The duration of the experiment was 49 days, with daily mortality monitoring. Anterior kidney samples (0.5 cm^3^) were collected from three live or moribund cohabitant fish at intervals of 0, 7, 14, 21, 28, 35, 42, and 49 days post-inoculation from one tank. Samples were stored in RNAlater (Life Technologies, Carlsbad, CA, USA) at −80 °C for further analysis. This study was approved by the ADL Bioethics Committee and conducted at the TEKBios fish trial center, ensuring ethical compliance in animal experimentation, which agrees with the recommendations made by the local National Agency for Research and Development, ANID, to ensure ethical compliance in animal experimentation (https://ayuda.anid.cl/hc/es/articles/4408576199060-Documentaci%C3%B3n-general-sobre-Bio%C3%A9tica-y-Bioseguridad, accessed on 4 September 2023).

### 4.6. Primer Design and Functional Quantitative Real-Time PCR (qPCR) Analysis

Primer design: the primer sets for the coding sequence of *ddx41*, *ifng*, *irf3*, *il1b*, and *tnfa* of *S. salar* and the coding sequence for the identification of *P. salmonis* and *R. salmoninarum* were designed using sequences retrieved from the NCBI database (GenBank accession number in Table S2) with the Primer-BLAST tool (https://www.ncbi.nlm.nih.gov/tools/primer-blast/) [81], using default parameters. Additionally, primers sequence from previous reports were used [76,82,83,84].

RNA extraction and cDNA synthesis: the total RNA extraction from fish tissues and cell cultures was performed using TRIzol (Life Technologies, Carlsbad, CA, USA) following the supplier’s instructions, and cDNA synthesis was performed with SuperScript III reverse transcriptase (Invitrogen, Waltham, MA, USA) with oligo dT from 2 µg of total RNA at 50 °C for 50 min.

Gene expression analysis: Real-time PCR was carried out on an Mx3005P quantitative PCR machine (Stratagene, La Jolla, CA, USA) using Brilliant II SYBR Green QPCR Master Mix reagent (Agilent Technologies, Santa Clara, CA, USA) to quantify gene expression. The reaction mixture was incubated for 10 min at 95 °C, followed by 40 cycles of 15 s at 95 °C, 1 min at 60 °C, and finally 15 s at 72 °C, 30 s at 58 °C, and 15 s at 95 °C. For each mRNA gene, expression was normalized to elongation factor-1α (*elf1a*). During melting curve analysis, once the amplification stage finished, amplicons were denatured at 95 °C for 15 s, followed by cooling at 0.1 °C/s up to 60 °C, capturing fluorescence in continuous mode.

The quantification was performed using the comparative Ct (2^−∆∆Ct^) method [85]. Several time points were considered for in vitro and in vivo assays, including one before infection kinetics began (time 0 or control). The primers used in qPCR are appended in Table S2. In all cases, each PCR was performed in triplicate and repeated with at least three independent samples.

### 4.7. Statistical Analysis and Graph

For in vitro gene expression, all data are shown as the mean ± standard error (SE). Assumptions of normality and homogeneity were tested for the detected variances. Differences were evaluated using one-way ANOVA and were considered significant at *p* < 0.05 [86]. Comparisons for each experimental point were carried out with the non-infected SHK-1 cell line (control). Statistically significant comparisons between infected cell lines at different hours post-infection (hpi) and the control are marked with asterisks, while non-significant comparisons are omitted.

For in vivo gene expression assays, three repeated experiments were conducted; non-parametric data distribution was considered using a Kruskal–Wallis analysis, and Dunn’s test was performed post hoc using GraphPad Software Inc. Comparisons were made for each experimental fish group at different days post-challenge (dpc) and the group of fish not challenged with *P. salmonis* (control). Data are presented as mean ± SE, with statistical significance defined as *p* < 0.05. Statistically significant comparisons between experimental times and the control are marked with asterisks, while non-significant comparisons are omitted. The plot was generated using GraphPad Prism (v. 9.2.0) and the ggplot2 package (v. 3.5.1) [87] in R software (v. 4.3.2) [88].

## 5. Conclusions

The current study offers crucial insights into the presence, structural functions, evolutionary patterns, and gene expression dynamics of the *ddx41* gene in *S. salar*. The heightened expression of *ddx41* during infections caused by *P. salmonis* and *R. salmoninarum* underscores its crucial role in initiating immune responses. Delving deeper into DDX41 signaling pathways in salmon will enrich our comprehension of teleost immunity, contributing to disease management in salmonid aquaculture. This study underlines the existence and expression significance of the DDX41 gene and protein in *S. salar* as a crucial immunological factor in salmonid health, as presented in this first report.

## Figures and Tables

**Table 1:** Organism, accession codes and blast results, from nucleotide and protein blast searches, against DDX41 of *H. sapiens* (AK222598.1/BAD96318.1).

Organism	Accession Number		BLASTn Results			BLASTp Results		
	Nucleotide	Protein	Query (%)	E-Value	Per. Ident. *	Query (%)	E-Value	Per. Ident. *
*H. sapiens*	AK222598.1	BAD96318.1	100	0.0	100	100	0.0	100
*R. norvegicus*	NM_001108046.2	NP_001101516.1	93	0.0	89.32	100	0.0	98.87
*G. gallus*	NM_001349708.2	NP_001336637.2	61	0.0	83.70	99	0.0	92.56
*D. rerio*	KR559928.1	ANB32180.1	83	0.0	78.93	100	0.0	85.69
*S. salar*	This study		91	0.0	78.61	100	0.0	83.92

* Per. Ident.: percent identity.

**Table 2:** Stereochemical quality check of models generated between *H. sapiens* and *S. salar* among domains and complete protein.

Organism	Domain	ERRAT	VERIFY 3D (Residues over Score Threshold)	PROCHECK (Error/Warning/Pass)
*S. salar*	Complete	84.6702	71.06%	4/3/2
*H. sapiens*	Complete	88.6029	63.90%	4/3/2
*S. salar*	Helicase ATP binding	94.2478	73.50%	0/3/6
*H. sapiens*	Helicase ATP binding	93.3628	83.76%	0/3/6
*S. salar*	Helicase C-terminal	96.1538	75.61%	2/3/4
*H. sapiens*	Helicase C-terminal	96.1538	79.88%	2/3/4

## Data Availability

The data presented in this study are available from the corresponding author upon request.

## Supplementary Materials

The following supporting information can be downloaded at: https://www.mdpi.com/article/10.3390/ijms25126346/s1. References [76,82,83,84] are mentioned in Supplementary Materials.

## References

[1] Sen R., Nayak L., De R.K. (2016). A review on host–pathogen interactions: Classification and prediction. Eur. J. Clin. Microbiol. Infect. Dis..

[2] Magnadóttir B. (2006). Innate immunity of fish (overview). Fish Shellfish Immunol..

[3] Wang Z., Zhang S., Tong Z., Li L., Wang G. (2009). Maternal transfer and protective role of the alternative complement components in zebrafish Danio rerio. PLoS ONE.

[4] Takeuchi O., Akira S. (2010). Pattern recognition receptors and inflammation. Cell.

[5] Yoneyama M., Fujita T. (2010). Recognition of viral nucleic acids in innate immunity. Rev. Med. Virol..

[6] Diacovich L., Gorvel J.P. (2010). Bacterial manipulation of innate immunity to promote infection. Nat. Rev. Microbiol..

[7] Villanueva-Cañas J., Ruiz-Orera J., Agea M.I., Gallo M., Andreu D., Albà M.M. (2017). New Genes and Functional Innovation in Mammals. Genome Biol. Evol..

[8] Armingol E., Officer A., Harismendy O., Lewis N.E. (2021). Deciphering cell-cell interactions and communication from gene expression. Nat. Rev. Genet..

[9] Mills E., Pultz I.S., Kulasekara H.D., Miller S.I. (2011). The bacterial second messenger c-di-GMP: Mechanisms of signaling. Cell Microbiol..

[10] Woodward J.J., Iavarone A.T., Portnoy D.A. (2010). c-di-AMP secreted by intracellular Listeria monocytogenes activates a host type I interferon response. Science.

[11] Parvatiyar K., Zhang Z., Teles R.M., Ouyang S., Jiang Y., Iyer S.S., Zaver S.A., Schenk M., Zeng S., Zhong W. (2012). The helicase DDX41 recognizes the bacterial secondary messengers cyclic di-GMP and cyclic di-AMP to activate a type I interferon immune response. Nat. Immunol..

[12] Dey B., Dey R.J., Cheung L.S., Pokkali S., Guo H., Lee J.H., Bishai W.R. (2015). A bacterial cyclic dinucleotide activates the cytosolic surveillance pathway and mediates innate resistance to tuberculosis. Nat. Med..

[13] Luecke S., Paludan S.R. (2017). Molecular requirements for sensing of intracellular microbial nucleic acids by the innate immune system. Cytokine.

[14] Li Y., Li H., Su N., Liu D., Luo R., Jin H. (2018). Molecular cloning and functional characterization of duck DDX41. Dev. Comp. Immunol..

[15] Jiang Y., Zhu Y., Liu Z.J., Ouyang S. (2017). The emerging roles of the DDX41 protein in immunity and diseases. Protein Cell.

[16] Perčulija V., Ouyang S., Tuteja R. (2019). Diverse Roles of DEAD/DEAH-Box Helicases in Innate Immunity and Diseases. Helicases from All Domains of Life.

[17] Omura H., Oikawa D., Nakane T., Kato M., Ishii R., Ishitani R., Tokunaga F., Nureki O. (2016). Structural and functional analysis of DDX41: A bispecific immune receptor for DNA and cyclic dinucleotide. Sci. Rep..

[18] Rocak S., Linder P. (2004). DEAD-box proteins: The driving forces behind RNA metabolism. Nat. Rev. Mol. Cell Biol..

[19] Kim K.H., Lee S., Park J.W., Jung H.S., Kim J., Yang H., Lee J.H., Lee D. (2021). Analysis of tissue-specific interferon regulatory factor 3 (IRF3) gene expression against viral infection in *Paralichthys olivaceus*. Dev. Reprod..

[20] Schröder M., Baran M., Bowie A.G. (2008). Viral targeting of DEAD box protein 3 reveals its role in TBK1/IKKepsilon-mediated IRF activation. EMBO J..

[21] Ma J.X., Li J.Y., Fan D.D., Feng W., Lin A.F., Xiang L.X., Shao J.Z. (2018). Identification of DEAD-Box RNA Helicase DDX41 as a trafficking protein that involves in multiple innate immune signaling pathways in a Zebrafish model. Front. Immunol..

[22] Zhang Z., Bao M., Lu N., Weng L., Yuan B., Liu Y.J. (2013). The E3 ubiquitin ligase TRIM21 negatively regulates the innate immune response to intracellular double-stranded DNA. Nat. Immunol..

[23] Quynh N.T., Hikima J., Kim Y.R., Fagutao F.F., Kim M.S., Aoki T., Jung T.S. (2015). The cytosolic sensor, DDX41, activates antiviral and inflammatory immunity in response to stimulation with double-stranded DNA adherent cells of the olive flounder, *Paralichthys olivaceus*. Fish Shellfish Immunol..

[24] Liu J., Huang Y., Huang X., Li C., Ni S.W., Yu Y., Qin Q. (2019). Grouper DDX41 exerts antiviral activity against fish iridovirus and nodavirus infection. Fish Shellfish Immunol..

[25] Gan Z., Cheng J., Hou J., Xia H., Chen W., Xia L., Nie P., Lu Y. (2020). Molecular and functional characterization of tilapia DDX41 in IFN regulation. Fish Shellfish Immunol..

[26] Qin X.-W., Luo Z.-Y., Pan W.-Q., He J., Li Z.-M., Yu Y., Liu C., Weng S.P., He J.G., Guo C.J. (2023). The Interaction of Mandarin Fish DDX41 with STING Evokes Type I Interferon Responses Inhibiting Ranavirus Replication. Viruses.

[27] Rozas M., Enríquez R. (2014). Piscirickettsiosis and *Piscirickettsia salmonis* in fish: A review. J. Fish Dis..

[28] Figueroa J., Cárcamo J., Yañez A., Olavarria V., Ruiz P., Manríquez R., Muñoz C., Romero A., Avendaño-Herrera R. (2019). Addressing viral and bacterial threats to salmon farming in Chile: Historical contexts and perspectives for management and control. Rev. Aquac..

[29] SERNAPESCA (2023). Sanitary Report with Sanitary Information of Freshwater and Sea Water. https://www.sernapesca.cl/app/uploads/2023/10/informe_sanitario_con_informacion_sanitaria_de_agua_dulce_y_mar_ano_2022.pdf.

[30] Madeira F., Park Y.M., Lee J., Buso N., Gur T., Madhusoodanan N., Basutkar P., Tivey A.R.N., Potter S.C., Finn R.D. (2022). The EMBL-EBI search and sequence analysis tools APIs in 2019. Nucleic Acids Res..

[31] Akira S., Uematsu S., Takeuchi O. (2006). Pathogen recognition and innate immunity. Cell.

[32] Medzhitov R. (2007). Recognition of microorganisms and activation of the immune response. Nature.

[33] Mogensen T.H. (2009). Pathogen recognition and inflammatory signaling in innate immune defenses. Clin. Microbiol. Rev..

[34] Mojzesz M., Klak K., Wojtal P., Adamek M., Podlasz P., Chmielewska-Krzesinska M., Matras M., Reichert M., Chadzinska M., Rakus K. (2020). Viral infection-induced changes in the expression profile of non-RLR DExD/H-box RNA helicases (DDX1, DDX3, DHX9, DDX21, and DHX36) in zebrafish and common carp. Fish Shellfish Immunol..

[35] Feng-Ying G., Mai-Xin L., Miao W., Zhi-Gang L., Xiao-Li K., De-Feng Z., Jian-Meng C. (2023). Nile tilapia DNA sensor STING is involved in the IFN-β and AP-1 signaling pathways in the immune response dependent on DDX41. Int. J. Biol. Macromol..

[36] Rauta P.R., Nayak B., Das S. (2012). Immune system and immune responses in fish and their role in comparative immunity study: A model for higher organisms. Immunol. Lett..

[37] Brocker C., Thompson D., Matsumoto A., Nebert D.W., Vasiliou V. (2010). Evolutionary divergence and functions of the human interleukin (IL) gene family. Hum. Genomics.

[38] Liu C., Chu D., Kalantar-Zadeh K., George J., Young H.A., Liu G. (2021). Cytokines: From Clinical Significance to Quantification. Adv. Sci..

[39] Secombes C.J., Wang T.H., Bird S. (2011). The interleukins of fish. Dev. Comp. Immunol..

[40] Zou J., Secombes C.J. (2016). The Function of Fish Cytokines. Biology.

[41] Sverdlov A.V., Rogozin I.B., Babenko V.N., Koonin E.V. (2005). Conservation versus parallel gains in intron evolution. Nucleic Acids Res..

[42] Almagro Armenteros J.J., Tsirigos K.D., Sønderby C.K., Petersen T.N., Winther O., Brunak S., Nielsen H. (2020). SignalP 5.0 improves signal peptide predictions using deep neural networks. Nat. Biotechnol..

[43] Laskowski R.A., MacArthur M.W., Moss D.S., Thornton J.M. (1993). PROCHECK: A program to check the stereochemical quality of protein structures. J. Appl. Crystallogr..

[44] Colovos C., Yeates T.O. (1993). Verification of protein structures: Patterns of nonbonded atomic interactions. Protein Sci..

[45] Zhang X., Brann T.W., Zhou M., Yang J., Oguariri R.M., Lidie K.B., Imamichi H., Huang D.W., Lempicki R.A., Baseler M.W. (2011). Cutting edge: Ku70 is a novel cytosolic DNA sensor that induces type III rather than type I IFN. J. Immunol..

[46] Andreou A.Z. (2021). DDX41: A multifunctional DEAD-box protein involved in pre-mRNA splicing and innate immunity. Bio. Chem..

[47] Krasteva P.V., Giglio K.M., Sondermann H. (2012). Sensing the messenger: The diverse ways that bacteria signal through c-di-GMP. Protein Sci..

[48] Schütz P., Karlberg T., van den Berg S., Collins R., Lehtiö L., Högbom M., Holmberg-Schiavone L., Tempel W., Park H.W., Hammarström M. (2010). Comparative structural analysis of human DEAD-box RNA helicases. PLoS ONE.

[49] Ouyang S., Song X., Wang Y., Ru H., Shaw N., Jiang Y., Niu F., Zhu Y., Qiu W., Parvatiyar K. (2012). Structural analysis of the STING adaptor protein reveals a hydrophobic dimer interface and mode of cyclic di-GMP binding. Immunity.

[50] Singh R.S., Vidhyasagar V., Yang S., Arna A.B., Yadav M., Aggarwal A., Aguilera A.N., Shinriki S., Bhanumathy K.K., Pandey K. (2022). DDX41 is required for cGAS-STING activation against DNA virus infection. Cell Rep..

[51] Tamassia N., Cassatella M.A. (2013). Cytoplasmic receptors recognizing nucleic acids and mediating immune functions in neutrophils. Curr. Opin. Pharmacol..

[52] Briard B., Place D.E., Kanneganti T.D. (2020). DNA Sensing in the Innate Immune Response. Physiology.

[53] Guimarães E.S., Marinho F.V., de Queiroz N.M.G.P., Antunes M.M., Oliveira S.C. (2021). Impact of STING Inflammatory Signaling during Intracellular Bacterial Infections. Cells.

[54] Jiang Y., Zhu Y., Qiu W., Liu Y.J., Cheng G., Liu Z.J., Ouyang S. (2017). Structural and functional analyses of human DDX41 DEAD domain. Protein Cell.

[55] Cheng Y., Liu Y., Wang Y., Niu Q., Gao Q., Fu Q., Ma J., Wang H., Yan Y., Ding C. (2017). Chicken DNA virus sensor DDX41 activates IFN-β signaling pathway dependent on STING. Dev. Comp. Immunol..

[56] Kadono M., Kanai A., Nagamachi A., Shinriki S., Kawata J., Iwato K., Kyo T., Oshima K., Yokoyama A., Kawamura T. (2016). Biological implications of somatic DDX41 p.R525H mutation in acute myeloid leukemia. Exp. Hematol..

[57] Zhang Z., Yuan B., Bao M., Lu N., Kim T., Liu Y.J. (2011). The helicase DDX41 senses intracellular DNA mediated by the adaptor STING in dendritic cells. Nat. Immunol..

[58] Altschul S.F., Gish W., Miller W., Myers E.W., Lipman D.J. (1990). Basic local alignment search tool. J. Mol. Biol..

[59] Larkin M.A., Blackshields G., Brown N.P., Chenna R., McGettigan P.A., McWilliam H., Valentin F., Wallace I.M., Wilm A., Lopez R. (2007). Clustal W and Clustal X version 2.0. Bioinformatics.

[60] Waterhouse A.M., Procter J.B., Martin D.M.A., Clamp M., Barton G.J. (2009). Jalview Version 2—A multiple sequence alignment editor and analysis workbench. Bioinformatics.

[61] Nylander J.A.A. (2004). MrModeltest v2. Program Distributed by the Author.

[62] Kumar S., Stecher G., Li M., Knyaz C., Tamura K. (2018). MEGA X: Molecular Evolutionary Genetics Analysis across computing platforms. Mol. Biol. Evol..

[63] Ronquist F., Teslenko M., Van der Mark P., Ayres D.L., Darling A., Höhna S. (2012). MrBayes 3.2: Efficient Bayesian phylogenetic inference and model choice across a large model space. Syst. Biol..

[64] Rambaut A. (2012). Figtree v1.4.4. https://github.com/rambaut/figtree/releases/tag/v1.4.4.

[65] Crooks G.E., Hon G., Chandonia J.M., Brenner S.E. (2004). WebLogo: A sequence logo generator. Genome Res..

[66] Letunic I., Khedkar S., Bork P. (2021). SMART: Recent updates, new developments, and status in 2020. Nucleic Acids Res..

[67] Jumper J., Evans R., Pritzel A., Green T., Figurnov M., Ronneberger O., Tunyasuvunakool K., Bates R., Žídek A., Potapenko A. (2021). Highly accurate protein structure prediction with AlphaFold. Nature.

[68] Mirdita M., von den Driesch L., Galiez C., Martin M.J., Söding J., Steinegger M. (2017). Uniclust databases of clustered and deeply annotated protein sequences and alignments. Nucleic Acids Res..

[69] Mirdita M., Steinegger M., Söding J. (2019). MMseqs2 desktop and local web server app for fast, interactive sequence searches. Bioinformatics.

[70] Mirdita M., Schütze K., Moriwaki Y., Heo L., Ovchinnikov S., Steinegger M. (2022). ColabFold: Making Protein folding accessible to all. Nat. Methods.

[71] Mitchell A.L., Almeida A., Beracochea M., Boland M., Burgin J., Cochrane G., Crusoe M.R., Kale V., Potter S.C., Richardson L.J. (2019). MGnify: The microbiome analysis resource in 2020. Nucleic Acids Res..

[72] Sali A., Blundell T.L. (1993). Comparative protein modeling by satisfaction of spatial restraints. J. Mol. Biol..

[73] Bowie J.U., Lüthy R., Eisenberg D. (1991). A Method to Identify Protein Sequences That Fold into a Known Three-Dimensional Structure. Science.

[74] Lüthy R., Bowie J.U., Eisenberg D. (1992). Assessment of protein models with three-dimensional profiles. Nature.

[75] Schrödinger, LLC (2015). The PyMOL Molecular Graphics System.

[76] Herrera V., Olavarría N., Saavedra J., Yuivar Y., Bustos P., Almarza O., Mancilla M. (2022). Complete lipopolysaccharide of *Piscirickettsia salmonis* is required for full virulence in the intraperitoneally challenged Atlantic Salmon, *Salmo salar*, model. Front. Cell. Infect. Microbiol..

[77] Dannevig B.H., Brudeseth B.E., GjØen T., Rode M., Wergeland H.I., Evensen Ø., Press C.M. (1997). Characterisation of a long-term cell line (SHK-1) developed from the head kidney of Atlantic salmon (*Salmo salar* L.). Fish Shellfish Immunol..

[78] Levican-Asenjo J., Soto-Rifo R., Aguayo F., Gaggero A., Leon O. (2019). Salmon cells SHK-1 internalize infectious pancreatic necrosis virus by macropinocytosis. J. Fish Dis..

[79] Yañez A.J., Silva H., Valenzuela K., Pontigo J.P., Godoy M., Troncoso J., Romero A., Figueroa J., Carcamo J.G., Avendaño-Herrera R. (2013). Two novel blood-free solid media for the culture of the salmonid pathogen *Piscirickettsia salmonis*. J. Fish Dis..

[80] Evelyn T.P.T. (1977). An improved growth medium for the kidney disease bacterium and some notes on using the medium. Bull. Off. Int. Epiz..

[81] Ye J., Coulouris G., Zaretskaya I., Cutcutache I., Rozen S., Madden T.L. (2012). Primer-BLAST: A tool to design target-specific primers for polymerase chain reaction. BMC Bioinform..

[82] Tapia D., Eissler Y., Torres P., Jorquera E., Espinoza J.C., Kuznar J. (2015). Detection and phylogenetic analysis of infectious pancreatic necrosis virus in Chile. Dis. Aquat. Org..

[83] Santibañez N., Vega M., Pérez T., Yáñez A., González-Stegmaier R., Figueroa J., Enríquez R., Oliver C., Romero A. (2020). Biofilm produced In Vitro by Piscirickettsia salmonis generates differential cytotoxicity levels and expression patterns of immune genes in the Atlantic Salmon cell line SHK-1. Microorganisms.

[84] Fredriksen B., Sævareid K., McAuley L., Lane M., Bøgwald J., Dalmo R. (2011). Early immune responses in *Atlantic salmon* (*Salmo salar* L.) after immunization with PLGA nanoparticles loaded with a model antigen and β-glucan. Vaccine.

[85] Pfaffl M.W. (2001). A new mathematical model for relative quantification in real-time RT-PCR. Nucleic Acids Res..

[86] Kim T.K. (2017). Understanding one-way ANOVA using conceptual figures. Korean J. Anesthesiol..

[87] Wickham H. (2016). ggplot2: Elegant Graphics for Data Analysis.

[88] RStudio Team (2020). RStudio: Integrated Development for R.

