# Supplementary material for: Discovery and Characterization of the ddx41 Gene in Atlantic Salmon: Evolutionary Implications, Structural Functions, and Innate Immune Responses to Piscirickettsia salmonis and Renibacterium salmoninarum Infections
Source: Int J Mol Sci. 2024 Jun 8;25(12):6346. doi: 10.3390/ijms25126346 (PMC11204154; doi:10.3390/ijms25126346)
Supplement: Supplementary file 1 [file ijms-25-06346-s001.zip › ijms-2992963-supplementary.pdf]

Supplementary Materials

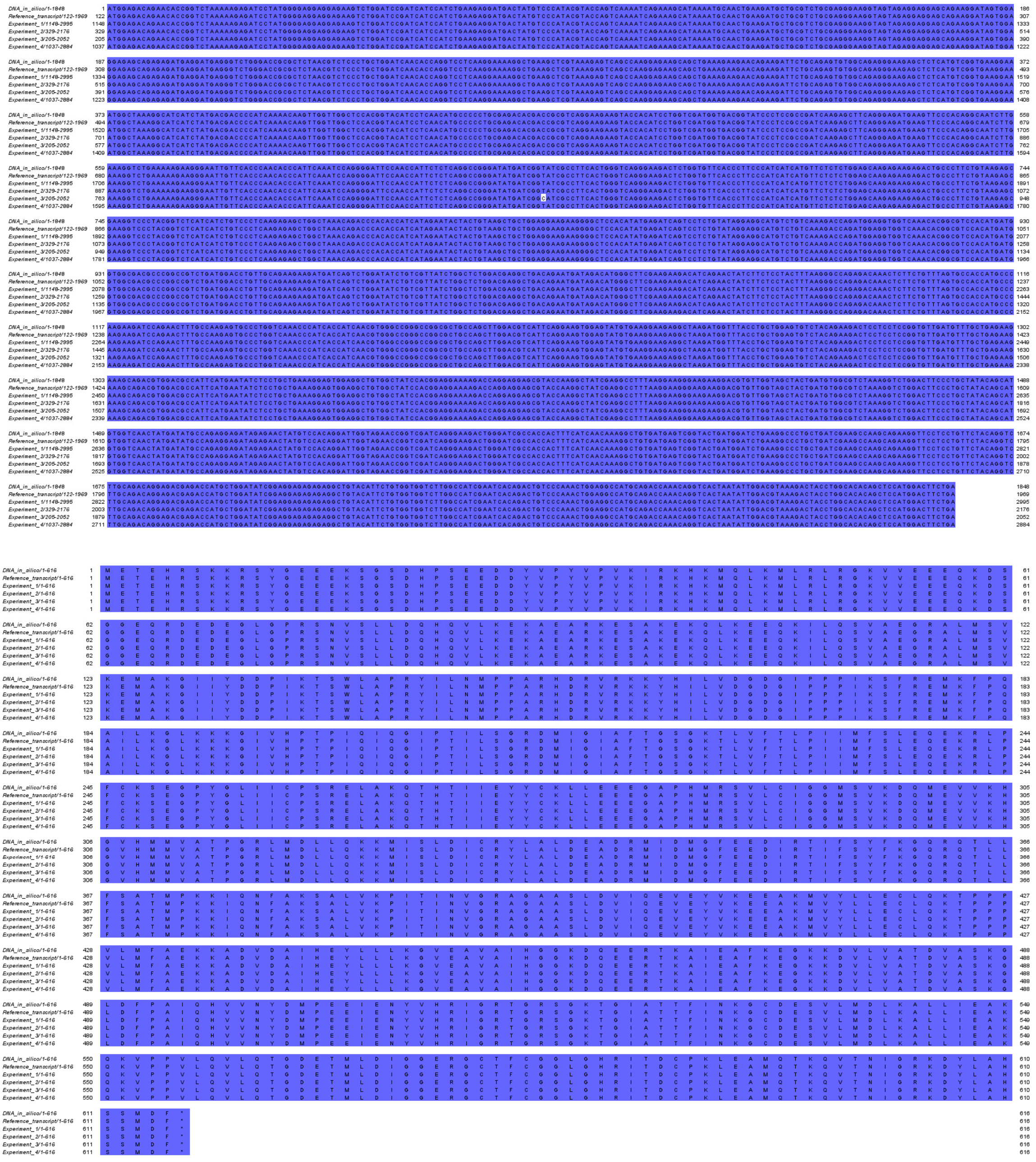

Figure S1. Alignment of *in-silico* DNA of *ddx41* gene, and its translation, against reference mRNA and four mRNA retrieved from Transcriptome Shotgun Assembly. Upper panel: Nucleotide sequences alignment. Bottom panel: Aminoacidic sequences alignment. Experiment 1 (Accession number GIYK01062020.1), Experiment 2 (Accession number GGAQ01023159.1), Experiment 3 (Accession number GEGX01092009.1), and Experiment 4 (Accession number GBRB01046301.1).

Table S1. Taxon and GenBank number used in phylogenetic analysis. Include percent of similarity and identity with respect to DDX41 sequence of *S. salar*

			**Percent similarity/identity *S. salar***	
	**Taxon**	**GenBank number**	**Nucleotide**	**Amino acid**
Fish	*Ctenopharyngodon idella*	MT259185	80.42	92.65
	Danio rerio	KR559928.1	81.57	92.16
	Salmo trutta	XM_029720241.1	98.76	99.84
	Oncorhynchus kisutch	XM_031797889.1	97.19	99.51
	Oncorhynchus tshawytscha	XM_042303535.1	96.97	99.51
	Oncorhynchus mykiss	XR_005035999.1	97.35	99.51
	Labrus bergylta	XM_020639468.2	82.38	99.84
	Paralichthys olivaceus	KJ934880.1	81.68	92.18
	Oreochromis niloticus	MN729497.1	81.30	92.51
	Micropterus salmoides	XM_038698792.1	82.06	92.83
	Perca flavescens	XM_028589760.1	82.06	93.32
	Epinephelus lanceolatus	XM_033632625.1	82.66	94.30
Amphibia	*Rhinatrema bivittatum*	XM_029583976.1	75.37	85.90
	Geotrypetes seraphini	XM_033920826.1	73.50	84.56
	Microcaecilia unicolor	XM_030212380.1	73.86	85.08
	Bombina bombina	XM_053717062.1	73.44	86.64
	Spea bombifrons	XM_053461876.1	74.67	85.19
	Nanorana parkeri	XM_018552392.1	73.97	85.33
	Bufo gargarizans	XM_044280801	74.21	86.42
	Bufo bufo	XM_040405458.1	73.93	86.04
Reptilia	*Hemicordylus capensis*	XM_053292688.1	75.74	84.89

	Gekko japonicus	XM_015412544.1	75.88	85.02
	Sphaerodactylus townsendi	XM_048491899.1	76.14	84.88
	Euleptes europaea	XM_056854428.1	75.28	84.53
	Lacerta agilis	XM_033140460.1	77.97	88.33
	Zootoca vivipara	XM_035105855.2	76.43	85.83
	Podarcis raffonei	XM_053377246.1	76.48	86.16
	Podarcis muralis	XM_028718488.1	76.48	86.16
	Anolis carolinensis	XM_008104964.2	74.43	84.99
	Pogona vitticeps	XM_020790536.1	75.50	85.74
Bird	*Myiozetetes cayanensis*	XM_050312154.1	77.56	86.88
	Zonotrichia albicollis	XM_005483745.2	77.62	86.88
	Vidua chalybeata	XM_053956756.1	77.40	87.04
	Parus major	XM_015642206.2	77.24	87.21
	Poecile atricapillus	XM_058848575.1	77.18	87.21
	Gallus gallus	NM_001349708.2	75.23	87.04
	Nipponia nippon	XM_009472159.1	76.89	87.40
	Anser cygnoides	XM_048080440.1	77.11	87.40
	Aythya fuligula	XM_032197202.1	77.38	87.23
	Anas platyrhynchos	KJ451069.2	77.33	87.23
	Calypte anna	XM_008498668.2	77.13	87.21
Mammalia	*Sorex araneus*	XM_004611428.2	75.81	84.82
	Echinops telfairi	XM_004696825.2	76.19	85.15
	Vicugna pacos	XM_006198933.3	74.80	84.96
	Bos taurus	NM_001082602.1	75.27	84.82
	Saimiri boliviensis boliviensis	XM_003936094.3	75.60	84.92

	Macaca thibetana thibetana	XM_050793205.1	75.49	84.82
	Homo sapiens	AK222598.1	75.32	84.82
	Castor canadensis	XM_020162697.1	75.49	84.98
	Mus musculus	NM_134059.2	75.05	85.15
	Rattus norvegicus	NM_001108046.2	75.38	84.98
	Gracilinanus agilis	XM_044661627.1	76.54	86.86

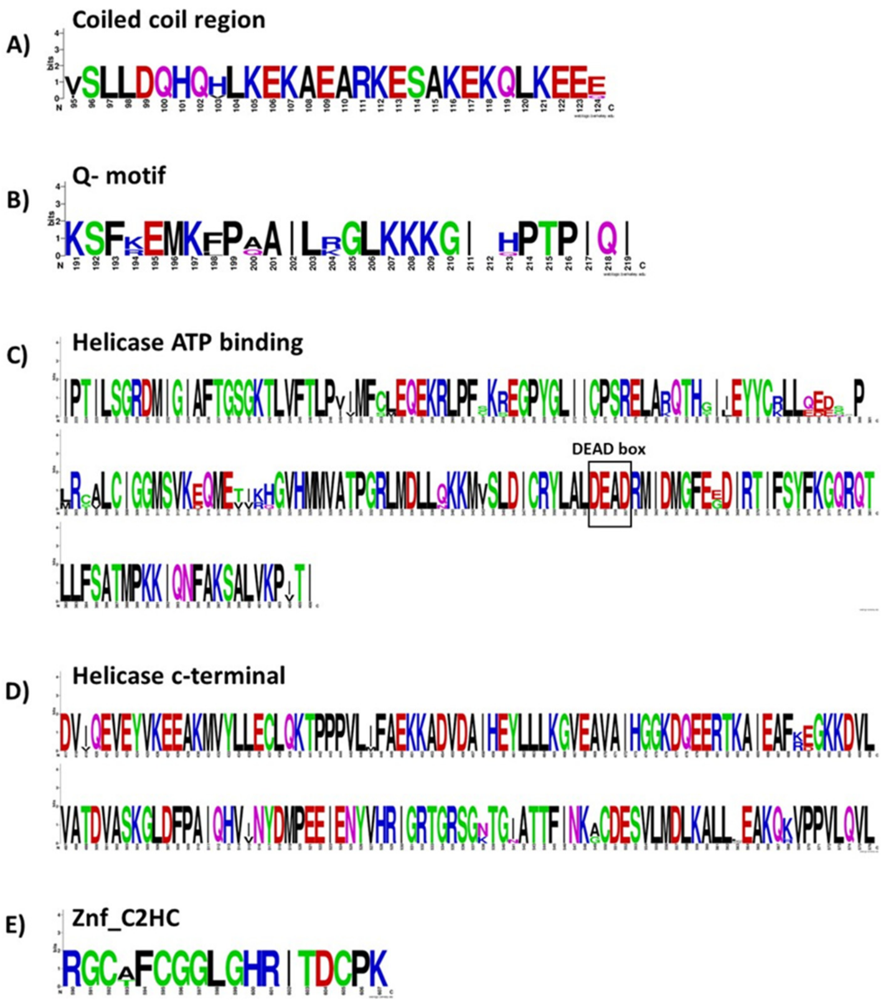

Figure S2. Sequence Logo generated of the DDX41 and similitude percent. The x-axis indicates the relative position of the amino acid and y-axis indicate . A) Coiled coil region (97.14%), B) Q-motif (100-89.66%), C) Helicase ATP binding, including DEAD box (97.03-94.05%), D) Helicase c-terminal (99.38-98.13%), E) Znf_C2HC (100-94.44%). The diagram was derived from multiple alignment of amino acid sequence of DDX41 of *S. salar, D. rerio, B. bufo, G. gallus, R. norvergicus* and *H. sapiens.*

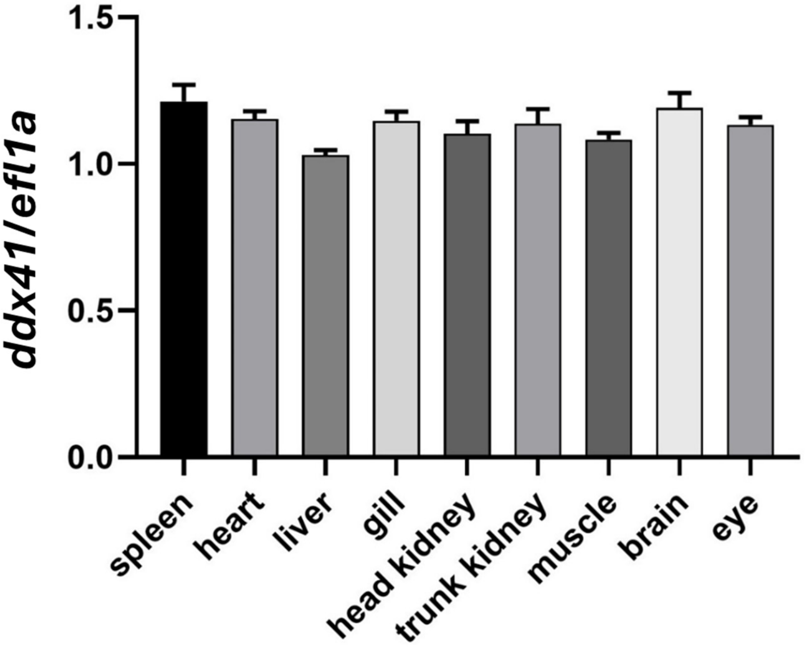

Figure S3. The expression of the *ddx41* gene examined in diverse organs/tissues of healthy *S. salar* by qPCR. Expression data were normalized to elongation factor-1α (*elf1a*) levels, with the liver serving as the control tissue. The data was presented as mean ± SE.

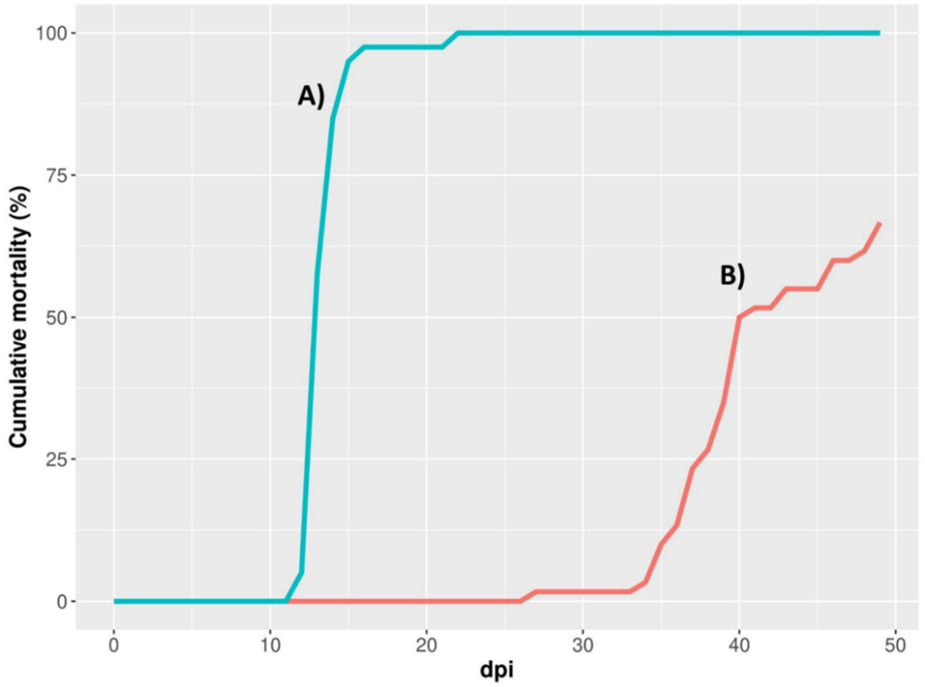

Figure S4. Cumulative Mortality of *S. salar* Infected by *P. salmonis* in Cohabitation with EM-90 strain (1 x 10^6 CFU/mL). A) Trojan and B) Cohabitant fish.

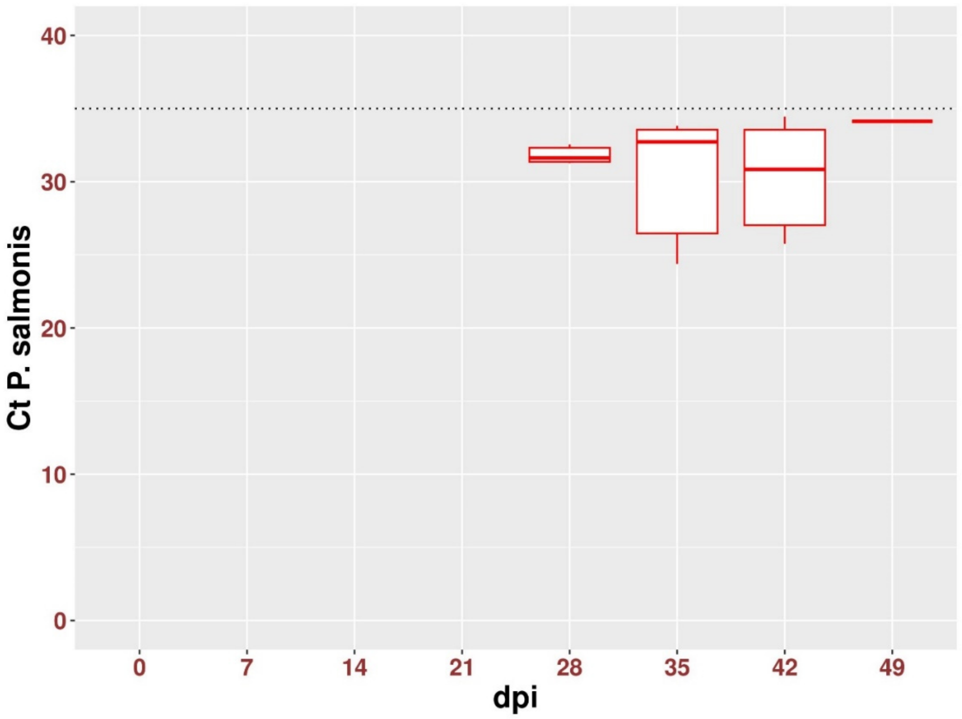

Figure S5. PCR detection of *P. salmonis* in kidney samples. DNA detection was performed by qPCR on anterior kidney samples from cohabitant fish. Each time point represents the results from 3 sampled fish. Dotted line refers to Ct threshold to consider a positive result (Ct < 35).

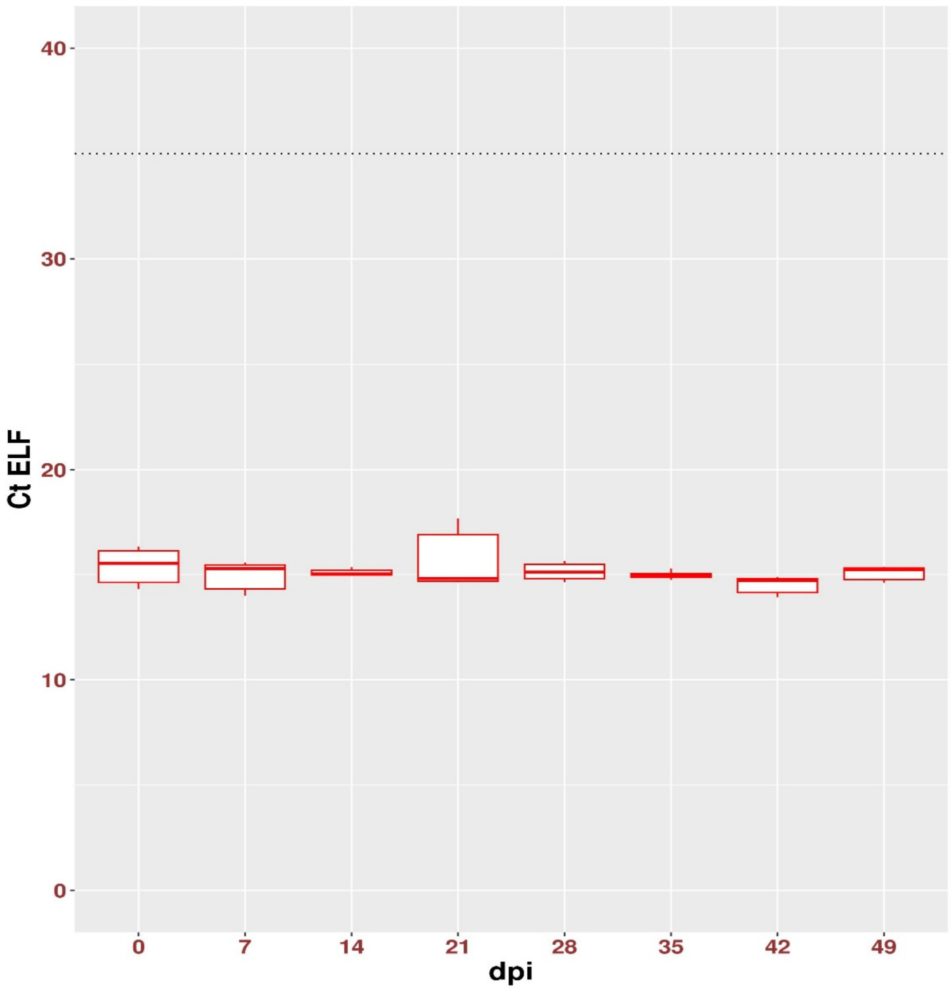

Figure S6. Ct values for *elf1a* during the *in vivo* infection kinetics. Dotted line refers to Ct threshold to consider a positive result (Ct < 35).

Table S2. List of primers used for qPCR.

Primer name	Forward sequence	Reverse sequence	Reference
P. salmonis	GCTGTGCCCAGAACTTTAG	GACCACTRCCTTTACCAAAC	[78]
R. salmoninarum	CACCACTAGCCCTCAACCAC	ACACATAACCGGGAGTGCAT	This work (CP000910.1)
elf1a	CCCCTCCAGGACGTTTACAAA	CACACGGCCCACAGGTACA	[88]
ddx41	GCCAGCTTGGACGTCATTCAG	CTGGTCTTTTCCTCCGTGGAT	This work (NM_001140327)
il1b	CAAGCTGCCTCAGGGTCT	CGCCACCCTTTAACCTCTCC	[89]
tnfa	CGTGGTGTCAGCATGGAAGA	AGTATCTCCAGTTGAGGCTCCATT	[90]
ifng	CGTGTATCGGAGTATCTTCAACCA	CTCCTGAACCTTCCCCTTGAC	This work (NM_001123558.1)
irf3	GCAGAGGGGATCTCAACCAC	GTGCCACATTGGAACGGTTG	This work (NM_001172282)
